# Genomic and functional analyses reveal *Pseudomonas granadensis* CT364 is a plant growth-promoting endophyte

**DOI:** 10.1186/s12866-025-04308-6

**Published:** 2025-10-10

**Authors:** Eva Cea Torrescassana, Maria del Carmen Montero-Calasanz, Marc Knight, Jem Stach, Thomas P. Howard

**Affiliations:** 1 School of Natural and Environmental Sciences, Newcastle University, Newcastle Upon Tyne, NE1 7RU UK; 2 IFAPA Las Torres - Andalusian Institute of Agricultural and Fisheries Research and Training, Junta de Andalucía, Cra. Sevilla-Cazalla, Km 12.2. 41200, Alcalá del Río, Seville, Spain; 3 Department of Biosciences, Durham University, South Road, Durham, DH1 3LE UK

**Keywords:** Endophyte, Plant growth-promoting bacteria, Chemotaxis, Plant-sensing

## Abstract

**Background:**

Plant-associated endophytes offer promising agricultural, environmental, and biotechnological applications. Despite their potential utility, difficulties in culturing these microorganisms under laboratory conditions have limited both their isolation and a comprehensive understanding of their biology, function, and ecological role. Against this background, *Pseudomonas granadensis* strain CT364—isolated from the olive tree rhizosphere—emerged as a potential endophyte of interest due to its cultivability and its ability to promote rooting across diverse species, including olive trees, rapeseed, mung bean and cowpea.

**Results:**

Genome Annotation and in silico predictions identified 564 genes linked to rhizosphere competence, plant colonisation and plant growth-promoting traits. Experimental findings confirmed the strain’s motility, capacity for biofilm formation, and ability to sense and respond to plant-derived signals. *P. granadensis* CT364 effectively colonises the rhizosphere, rhizoplane, and internal tissues of Arabidopsis, confirming its endophytic nature without exhibiting any pathogenic traits. Inoculation experiments demonstrated significant effects on root architecture and increases in plant biomass and rosette area. Notably, these benefits were retained under salinity and osmotic stress, underscoring its plant growth-promoting ability. Finally, both genome analysis and experimental tests confirmed its resistance to osmotic stress and heavy metal toxicity, highlighting the strain’s ability to survive in difficult environments.

**Conclusions:**

The integration of genomic insights and experimental validation supports the conclusion that *P. granadensis* CT364 is a plant growth-promoting endophytic bacterium. Its ability to enhance plant development under both optimal and stressful conditions, combined with its ability to colonise Arabidopsis and non-pathogenic nature, positions this strain as a potential bioinoculant for sustainable agriculture. Furthermore, the identification of specific genes related to plant sensing and colonisation, and its genetic tractability, open avenues for exploring underlying mechanisms of plant–microbe interactions. In summary, *P. granadensis* CT364 therefore not only holds potential for improving crop performance under challenging environmental conditions but also offers a valuable model for the study of beneficial plant–bacterial symbiosis.

**Supplementary Information:**

The online version contains supplementary material available at 10.1186/s12866-025-04308-6.

## Background

Plant growth-promoting bacteria (PGPB) directly enhance plant growth by producing bioactive compounds that alleviate stress [91, 104, 113], improving nutrient uptake through siderophore production or phosphate solubilisation [5, 156], and inducing developmental changes in plants via hormone secretion [48, 85]. In addition, they indirectly alleviate biotic stresses by competing within their ecological niche [112, 145], either through the biosynthesis of antimicrobial or insecticidal compounds [49, 138], or by activating plant induced systemic resistance [54].

PGPBs adopt various lifestyles, including free-living, rhizospheric or endophytic forms. Endophytes are particularly valuable for crop production because of their intimate association with their plant host and its phytopathogens. This close relationship enables endophytic PGPBs to enhance both plant growth promotion and biocontrol performance [8, 34, 79, 107]. Successfully identifying effective plant-colonising and growth-promoting bacterial endophytes, however, remains challenging [87, 159]. Cultivation difficulties and the lack of robust characterisation methods limit our understanding of plant–microbe interaction specificity and endophyte performance [1, 143]. The application of endophytic PGPB to improve plant productivity is also hindered by their survival in the competitive and chemically complex rhizosphere – a critical prerequisite for successful colonisation. In this environment, endophytes are exposed to plant defence compounds, microbial competition and abiotic stresses [56, 151]. Consequently, rigorous screening and selection methods are essential to identify appropriate candidate PGPBs for agricultural application. Recent advances in microscopy and omics-based approaches have significantly improved the discriminatory power of functional analyses facilitating more accurate characterisation of endophytic strains and host interactions [11, 131].

Root colonisation by bacteria is governed by the dynamic chemical and physical nature of the plant rhizosphere. Chemotaxis is important, allowing bacteria to sense root exudates and initiate a motile response towards them [79, 82, 122]. These responses are activated by root exudates and other environmental signals that regulate flagella- and type IV pilus-driven motility, biofilm formation, and exopolysaccharide (EPS) production [53, 73, 144, 158, 176, 182]. Root surface exploration and attachment are determined by biofilm formation and filamentation [36, 46, 80, 116, 129]. For instance, *Agrobacterium tumefaciens* shows filamentation in proximity to plant roots, whereas *Pseudomonas fluorescens* tends to filament under nutrient limitation or when in contact with surfaces [58, 153].

The pseudomonads hold significant importance in agriculture due to their ecological and lifestyle variability, coupled with multiple modes of promoting plant health, including biocontrol capabilities and direct plant growth promotion [125, 145]. Beneficial strains include *P. fluorescens* Pf-01, which serves as a model organism for root colonisation and plant growth promotion via soil pollutant degradation and plant disease suppression via antimicrobial and siderophore synthesis [62]. Similarly, the plant growth-promoting rhizobacteria *P. simiae* WCS417, *P. putida* KT2440 and *P. protegens* elicit induced systemic resistance, hormone production and siderophore synthesis [42, 44, 45]. The endophytic strain *P*. *stutzeri* A1501 meanwhile contributes to plant growth promotion through nitrogen fixation [121, 166] while *P. fluorescens* PICF7 acts as a biocontrol and plant growth-promoting endophyte [97, 111]. In some cases, synergistic interactions are observed. *P. fluorescens* F113, for example, improves the PGP performance of *Azosphirillum brasilense* [42]. Despite these many and varied beneficial strains, pathogenic strains like *P. syringae* pv. *tomato* (*Pst*) strain DC3000 and *P. aeruginosa* PAO1, can also be found within this diverse group [10, 140].

*P. granadensis* is a recently delineated species that has not previously been documented as a plant endophyte. The strain designated CT364 was isolated from Olive (*Olea europaea*) roots in Mairena del Aljarafe, Sevilla (Spain) [110]. Initial phenotypic characterisation revealed the ability of this strain to colonise and promote the growth of olive cuttings, induce root elongation in rapeseed plants (*Brassica napus*) and promote root development in mung bean (*Vigna radiata* L.) and cowpea (*V. unguiculata*), suggesting a potentially broad host range. Very recently *P. granadensis* R4-79, isolated from the desert plant *Ifloga spicata*, was shown to endophytically colonise tomato (*Solanum lycopersicum*) [4], further expanding the ecological relevance of this species. *P. granadensis* CT364 exhibits classical PGP traits including phosphate solubilisation, siderophore production and the synthesis of plant hormones [110]. To further explore the potential for *P. granadensis* to act as a PGPB we previously reported its complete genome sequence [35]. Genomic analysis of CT364 revealed a distinctive combination of traits that extend beyond those commonly reported in well-studied PGP Pseudomonas species such as *P. fluorescens*, *P. simiae*, and *P. putida*. These include an expanded suite of biosynthetic gene clusters—such as those for fragin, lokisin, and hydrogen cyanide—as well as multiple secretion systems (Type IV and VI) and stress-tolerance loci associated with osmotic and oxidative resilience. These features suggest enhanced biocontrol potential and ecological fitness, particularly under abiotic stress. Given that sequencing-based studies may disconnect genomic predictions from biological function [83], the goal of the work presented here was to combine comparative genome and genome mining analysis with in vitro and in planta experimental validation to characterise the endophytic and PGP traits of *P. granadensis* CT364. Arabidopsis provides an excellent model in which to investigate these traits, enabling analyses of bacterial colonisation, stress mitigation, and plant growth promotion—analyses that are difficult to perform in crops such as olive due to their long generation times and limited suitability for controlled inoculation studies. Moreover, the use of Arabidopsis establishes a platform for future investigations in which both the plant and the microbe are genetically defined and experimentally tractable. Our results demonstrate that *P. granadensis* CT364 can colonise the model plant Arabidopsis and that it exhibits traits essential for an endophytic lifestyle, including motility, plant compound sensing, and biofilm formation. The genome also contains genes associated with abiotic stress tolerance, hormone synthesis, alleviation of plant stress, siderophore production, synthesis of antimicrobial and insecticidal compounds. Our experimental validation using Arabidopsis confirmed *P. granadensis* CT364’s PGP-benefits, while microbiological analysis demonstrated this strain possesses high tolerance to salinity, osmotic stress and heavy metals. Together, the integration of genomic insights and experimental data supports the designation of *P. granadensis* CT364 as a PGP endophytic bacterium.

## Methods

### Microbial growth

*Pseudomonas granadensis* CT364 was grown in TSB or on TSB agar (TSA) plates at 28°C. BD Bacto Tryptic Soy Broth (TSB) was from ThermoFisher Scientific, USA. For all the assays, a starter culture was obtained by overnight incubation in TSB with shaking at 180 rpm and a 1:10 dilution for 4 h for culture synchronisation (i.e. to ensure all cultures were actively growing). *Escherichia coli* DH5α *λpir* was grown in LB or in LB agar (LBA) plates at 37°C. LB Miller was from Merk (Germany).

### Creating a GFP-expressing P. granadensis CT364 strain

A CT364 strain with constitutive GFP-expression was developed via suicide plasmid (*gfp*-*pSNW2*) genomic insertion [169] downstream of *glmS*, a neutral intergenic region in *Pseudomonas* [84]. Two insertion site-homology arms were amplified via Phusion U Hot Start (Thermo Scientific, USA) (Table 1) and cloned and inserted into the *pSNW2* plasmid [169], using USER Enzyme (NEB, USA), following the manufacturer’s instructions. The *E. coli* strain DH5α *λpir* harbouring the pSNW2-*glmS* plasmid was cultured in LB broth supplemented with kanamycin (50 μg/mL) for plasmid replication and extraction (QIAprep Spin Miniprep Kit; Qiagen, Germany). 500 ng of the replicated plasmid were then electroporated into the *Pseudomonas* strain [98]. Briefly, an overnight cultured was washed three times in sterile 300 mM sucrose, and competent cells were electrically pulsed at 2.5 kV, 12.5 kV/cm, 25 μF, 200 Ω, pulse time: 4–5 ms (Micropulser, Bio-Rad, USA). After electroshock, 1 mL of TSB was added to the cuvette for resuspension and culture under standard conditions for three hours. Finally, a 100 μL culture aliquot was plated on selective TSA plates containing kanamycin (100 μg/mL). The presence of the pSNW2-*glmS* suicide plasmid was confirmed by antibiotic resistance and green fluorescence using a transilluminator. The selected transformants, named as CT364-pSNW2, were preserved in 25% glycerol at −80°C.

**Table 1:** Primers used in this study. All oligonucleotides were ordered Lyophilised as standard 25 nmol from Integrated DNA Technologies (IDT, USA). On their arrival, oligos were resuspended in nuclease-free distilled water to 100 μM and stored at −20 °C

Name	Sequence
HA1_*glm*S_FW	AGATCCUGCATTTGCACCGTGTAAAACC
HA1_*glm*S_RV	ATGGGCACGAAUGGGCGTTGCGGGATTATC
HA2_*glm*S_FW	AGGCATUCGGTGGACGTCATTAAATAGAA
HA2_*glm*S_RV	AGGTCGACUGTGGAAGAGTTGCGTCGC

### Arabidopsis seed sterilisation and germination

*Arabidopsis thaliana* Col-0 seeds were surface sterilised overnight in a desiccator using chlorine vapours generated by combining 4 mL of concentrated HCl (37%) with 100 mL of commercial bleach. Seeds were sown in square Petri dishes (12 × 12 cm) of half-strength Murashige and Skoog media (Murashige and Skoog, 1962; Merk, Germany) supplemented with 1% (w/v) sucrose (Merk, Germany) 1% (w/v) agar (Merk, Germany) (^1^/_2_ MS). Seeds were stratified by storage at 4 °C in the dark for three days. Seeds were germinated via vertical incubation in a plant growth chamber (PRC 1200 WL, Hettich, Netherlands) in the following conditions: 22 °C, 70% humidity, 16/8 h photoperiod, at 100 μmol m^−2^ s^−1^.

### Arabidopsis growth assays

Seven-day-old Arabidopsis seedlings grown in ½ MS plates were transferred either to fresh ½ MS plates supplemented with 100 mM NaCl for in vitro plant growth promotion assays, or to Jiffy-7 41 mm peat pellets (Jiffy products, Plant Products Ltd., Canada) for evaluation under soil-mimicking axenic conditions (Figure S1). The bacterial inoculum (OD_600_ = 0.02) was prepared from a synchronised bacterial culture, washed three times by centrifugation at 3,000 g and resuspension in 10 mM MgSO_4_ sterile solution. For the axenic soil-mimicking assays (peat pellets), plants were inoculated with 2 mL of bacterial inoculum or 10 mM MgSO_4_, as a negative control. Plants were watered every three days with sterile deionised water. Salinity was simulated by single irrigation with a 100 mM NaCl solution one week after bacterial inoculation. Four-week-old plants were collected for measurement of whole plant fresh weight, rosette fresh and dry weight, and total rosette surface area by ImageJ [147]. The assay (*n* = 25 replicates) was repeated three times. For the in vitro assays, 7-day-old seedlings were primed with CT364 inoculum or 10 mM MgSO_4_ as a negative control. After one hour, the seedlings were transferred to fresh ½ MS and ½ MS supplemented with 100 mM NaCl Petri dishes. Three-week-old plants were photographed. The assay included *n* = 20 replicates and was repeated three times All experiments were conducted in a plant growth chamber under the conditions described above.

### Visualisation of rhizosphere colonisation

Adjacent, or in-distance, inoculation was used for the in vivo visualisation of rhizosphere colonisation. Seven-day-old Arabidopsis seedlings were placed in a fresh ½ MS 3% sucrose Petri plates. A CT364-pSNW2 synchronised overnight bacterial culture, which was subsequently washed three times in 10 mM MgSO_4_ and adjusted to an OD_600_ = 0.5, was added to the plates in 50 µL aliquots, 5 cm from seedling roots. The non-contact bacterial inoculation effects were photographed two weeks after inoculation under visible and UV Light. The assay included 15 replicates, and the experiment was repeated three times. Plants were grown in a plant growth chamber under the conditions described above.

### Rhizosphere and endosphere colonisation assay with confocal laser scanning microscopy

To analyse the ability of CT364 isolate to colonise plants, 7-day-old Arabidopsis seedlings were inoculated with CT364-pSNW2 (OD_600_ = 0.02) or 10 mM MgSO_4_ as a negative control. Plants were grown in a plant growth chamber under the conditions described above. Three-week-old seedlings were then left unwashed or washed with ethanol for 1 min and washed three times with sterile distilled water to remove unbound bacteria. The resulting samples were mounted with a drop of Citifluor AF1 anti-fading solution (AF1-25, USA) and visualised using a confocal microscope (SP8 TCS, Leica, Germany) via two capture channels and excitation laser wavelengths of 488 nm and 568 nm. Images were processed via Fiji software [146].

### Motility assays

Swimming and swarming motility assays were performed on 0.3% or 0.5% agar plates of 0.1 × Cook’s Cytophaga (CC) (0.2% tryptone) [40]​, respectively​. A synchronised overnight culture was washed three times in 0.1 × CC and adjusted to a cell density corresponding to OD_600_ = 1. For swimming assays, plate inoculation was performed by dipping a sterile 10 μL pipette tip into the cell suspension and piercing the agar plate until halfway through. For swarming assays, plates were inoculated by adding 1 µL of the cell suspension to the agar surface. In both cases, the plates were incubated upright at 28 °C for 72 h before the phenotype was observed and photographed.

### Chemotaxis assay

Chemotaxis was evaluated via a drop assay [57, 65]. Briefly, 40 mL of CT364 overnight synchronised culture was pelleted and resuspended in 12 mL of chemotaxis buffer (100 mM potassium phosphate, 20 μM EDTA, pH 7.0). After mixing with 3 mL of 1% hydroxypropylmethylcellulose (4,000 cP viscosity in 2% aqueous solution), the bacterial suspension was transferred to a Petri dish, and a 0.5 mM 10 μL drop of the compounds to test (arabinose, cellobiose, glucose, fructose, mannitol, sucrose, spermidine, aspartate, citrate, fumarate, glutamate, malate, succinate, oxalate, xylose) (Merk, Germany) and 15, 18 or 21-day Arabidopsis root exudates were added to the centre of the plate. Exudate collection was conducted specifically at this development because Arabidopsis roots are known to release a wide variety of phytochemicals at this stage (18–21) [14, 117]. An extra time point, using 15-day-old seedlings was used to evaluate potential differences depending on the developmental stage. After a 30-min incubation at room temperature, the plates were observed and photographed. The appearance of a bacterial halo and a clear zone was considered a positive chemotactic response towards the chemoattractant component.

### Induced biofilm formation assay using crystal violet staining

Root exudates were collected from 15-, 18- and 21-day-old seedlings grown in 24-well plates (Thermo Fisher Scientific, USA) containing 1 mL of ½ MS liquid medium [14, 15]​ thereby allowing uniform plant growth with minimal medium use, reduced cross-contamination risk, and reproducible imaging and sampling. To achieve this, twelve 7-day-old Arabidopsis seedlings, grown on ½ MS plates (following the previously described procedures), were placed in each well and incubated on an orbital shaker at 90 rpm with a photoperiod of 16/8 h light/dark at 22 °C for 4, 8 or 11 days. The seedlings were subsequently rinsed with sterile distilled water and transferred to fresh 24-well plates with 1 mL of sterile deionised water for a 3-day incubation, under the same conditions. The exudates were collected, filtered through 0.45 µm nylon filters (Millipore, USA) and aliquoted into 1.5 mL tubes (Eppendorf, Germany) and stored at −80 °C.

Biofilm formation was quantified via the crystal violet-based method [41, 136]​. A synchronised overnight culture was washed and resuspended in TSB to a final cell density of OD_600_ = 0.01. Aliquots of 90 μL were poured into a 96-well plate. Before static incubation for 4 h, 10 μL of 1 × 15-, 18-, 21- Arabidopsis root exudates, or sterile distilled water as a negative control, were added to the wells. This incubation time was selected following preliminary experiments indicating that biofilm formation peaked at this time. The cell density (OD_600_) was measured after incubation for data standardisation. The plates were inverted for washing three times with 200 μL of PBS (8 g/L NaCl, 0.2 g/L KCl, 1.44 g/L Na_2_PO_4_) per well. After drying, the biofilm cells were fixed with 150 μL of methanol and incubated for 20 min. After this time, the plates were flicked and dried overnight. The biofilms were stained with 125 μL of 0.1% crystal violet for 15 min and washed by three immersions in ddH_2_O. After drying, biofilms were solubilised with 150 μL of 30% glacial acetic acid. After 10 min of incubation, the optical density was measured at 570 nm (OD_570_). The following formula was used to calculate the Specific Biofilm Formation Index: SBF = (Sample OD_570_ – Average OD_570_ of negative controls)/Sample OD_600_. The experiments were performed with three biological replicates.

### Biofilm visualisation via confocal laser scanning microscopy

The biofilm formation of CT364-pSNW2 (GFP-expressing) was visualised using confocal laser scanning microscopy. A synchronised overnight culture was washed with M63 (2 g/L (NH_4_)_2_SO_4_, 13.6 g/L KH_2_PO_4_, 0.5 mg FeSO_4_−7H_2_O, 24.6 MgSO_4_−7H_2_O, 20 g/L glucose) and adjusted to an OD_600_ of 0.01. Then, 8 mL of this bacterial culture was added to a 50-mL tube (Falcon, USA) where a sterile coverslip was submerged for static incubation at 28 ºC. After one week, the coverslip was fixed to a glass slide using an adhesive spacer (0.05 mm iSpacer, SUNJin Lab, Taiwan) and AF1-25 anti-fading solution (Citifluor, USA). The biofilms were visualised with an SP8 TCS confocal microscope (Leica, Germany), with excitation at 488 nm and emission detection ranging from 490 to 580 nm. Images were processed using Fiji software [146].

### Hydrogen cyanide (HCN) production assay

The bacterial production of HCN was determined via colorimetric reaction via qualitative method [16]. Sterile Whatman filter paper strips were dipped into a solution of 0.5% pipric acid and 2% Na_2_CO_3_ and attached to the Petri dish lids. A 50 µL aliquot of *P. granadensis* CT364, *P. fluorescens* ACTT 13525^ T^ and *E. coli* DH5⍺ overnight cultures was spread onto TSA plates. *P. fluorescens* ACTT 13525^ T^ was used as a positive control for HCN production, while *E. coli* DH5⍺ cultures and sterile distilled water were used as negative controls. The plates were sealed with parafilm and incubated at 28°C for 72 h before being photographed. The colorimetric change of the paper strips from yellow to red brown indicated HCN production.

### Antimicrobial activity: Bacillus sp. reporter strain assay

The antimicrobial activity and potential mode of action of *P. granadensis* CT364 were screened by plug assay using *B. subtilis* strains carrying *lacZ* fusions to antibiotic-responsive promoters (*ϕ*105*, fabHA, ypuA, helD* and *liaI)* [81]. Overnight cultures of the reporter strains were grown in nutrient broth at 30 °C and 200 rpm, mixed with melted nutrient agar (1:1, v/v), and supplemented with 100 μg/mL X-Gal prior to plating.

Agar plugs of *P. granadensis* CT364 and *P. fluorescens* ACTT 13525^ T−^ grown on TSA were placed on the reporter strain plates. Antimicrobials known to induce promoter expression— nalidixic acid (25 μg/mL, Thermo Fisher Scientific) for *ϕ*105, triclosan (1 μg/mL, Merck) for *fabHA*, cefoxitin (25 μg/mL, Merck) for *ypuA*, rifampicin (50 μg/mL, Melford) for *helD*, and bacitracin (500 μg/mL, Merck) for *liaI* —were used as positive controls by applying 10 μL to sterile paper strips (Merk, Germany). Plates were incubated at 28 °C for 48 h. Zones of inhibition and blue halos around the plug or strip were measured to evaluate antibacterial activity and identify potential mechanisms of action.

### Bioinformatic analysis

#### Comparative genome analysis

Blast atlases were generated using the GVIEW JAVA package software [120] by performing tblastx analyses with the default parameters. The strains included *P. granadensis* CT364 (NZ_CP069352.1), and its two closest strains *P. granadensis* LMG 27940^ T^ type strain (NZ_LT629778.1) and *P. moraviensis* LMG 24280^ T^ (NZ_LT629788.1), known PGP strains (*P. fluorescens* Pf-01 (NC_007492.2), *P. simiae* WCS417 (NZ_CP007637.1), *P. stutzeri* A1501 (NC_009434.1), *P. chlororaphis* PA23 (NZ_CP008696.1), *P. protegens* Pf-5 (NC_004129.6), *P. putida* KT2440 (NC_002947.4)), and pathogenic strains (*P. aeruginosa* PAO1 (NC_002516.2), *P. syringae pv. tomato* DC3000 (NC_004578.1). The *P. granadensis* CT364 genomic islands, identified in silico by Islandviewer4, were also included in the BLAST alignment.

The pan-genome study was performed using BPGA (Bacterial Pan Genome Analysis tool; ​[37]) and compared the genomes of *P. granadensis* CT364 (NZ_CP069352.1) and its closest relatives (*P. koreensis* LMG 21318^ T^ ​(NZ_LT629687.1), *P. moraviensis* LMG 24280^ T^ (NZ_LT629788.1), described PGP *Pseudomonas* (*P. fluorescens* Pf-01 (NC_007492.2), *P. fluorescens* SBW25 ​(NC_012660.1), *P. protegens* Pf-5 (NC_004129.6), *P. putida* BIRD-1 ​(NC_017530.1), *P. putida* KT2440 (NC_002947.4), *P. putida* W619 ​(NC_010501.1), *P. simiae* WCS417 (NZ_CP007637.1) and the pathogen *P. aeruginosa* PAO1 ​(NC_002516.2). The pre-processing step for sequence clustering was done using the standard USEARCH tool [52].

#### Genome annotation

Genome annotation was performed using the Prokaryotic Genome Annotation Pipeline (PGAP) version 4.8 [66, 92]. Other servers were used to complete and compare the annotation output, such as the Rapid Annotation using Subsystem Technology (RAST) server v2.0 [12] and Prokka v1.8. [148] with default parameters. The BLAST search algorithm [27], Gene Ontology [9] and Interpro [119] were used to investigate the function of the hypothetical proteins predicted by the annotation. The virulome was identified using the Virulence Factor Database (VFDB [95]​, genomic islands with IslandViewer v4 [22] and the prophage sequences with PHASTER [7]. AntiSMASH [25] and polyketide synthetases product predictor [6] were used to predict the secondary metabolite clusters and possible products. The mobile elements or insertion sequences were identified using the IS Finder database [152] and blast searches with the default E-value threshold of e-10. The output of these software and the genomic data were used for the exhaustive description of the referenced genes related to plant growth promotion and the identification of beneficial genomic traits for an agricultural application.

### Methyl Accepting Protein (MCP) bioinformatic analysis

A total of 38 MCPs predicted from the *P. granadensis* CT364 genome were compared to the MCP repertoire in the Sanchis-López et al. database using BLASTn (BLAST + v.2.9.0) [33]. Each MCP was annotated based on the best match from the database, including relevant metadata associated with the MCP. For MCPs with identified sensor domains, the Degree of Plant Specificity (DPS) for the corresponding sensor domain subfamily was provided. To incorporate ecological context, the 38 predicted MCPs were mapped against the Genomic Catalog of Earth’s Microbiomes (GEM) [59] using BLASTx (BLAST + v.2.9.0) [33]. Prior to this, all metagenomic sequences containing the MCPsignal domain (PFAM: PF00015) [59] were extracted from the GEM catalog using HMMER (v.3.2.1, e-value 0.0001) [124]​. The best match for each MCP from *P. granadensis* CT364 was annotated with its corresponding ecological information.

### Statistical analysis

Experimental data were analysed by one-way or two-way analysis of variance (ANOVA) and statistically significant differences were considered between the control and bacterial-treatments at *p*-value < 0.05 level using Tukey’s Honestly Significant Differences (HSD) test from the *dplyr* R studio package v1.0.7 [177]. Standard deviation was calculated for all mean values.

## Results

### *P. granadensis* CT364 is a motile, plant-sensing, biofilm-forming strain able to endophytically colonise Arabidopsis

#### Motility and chemotaxis analysis

**Fig. 1:**
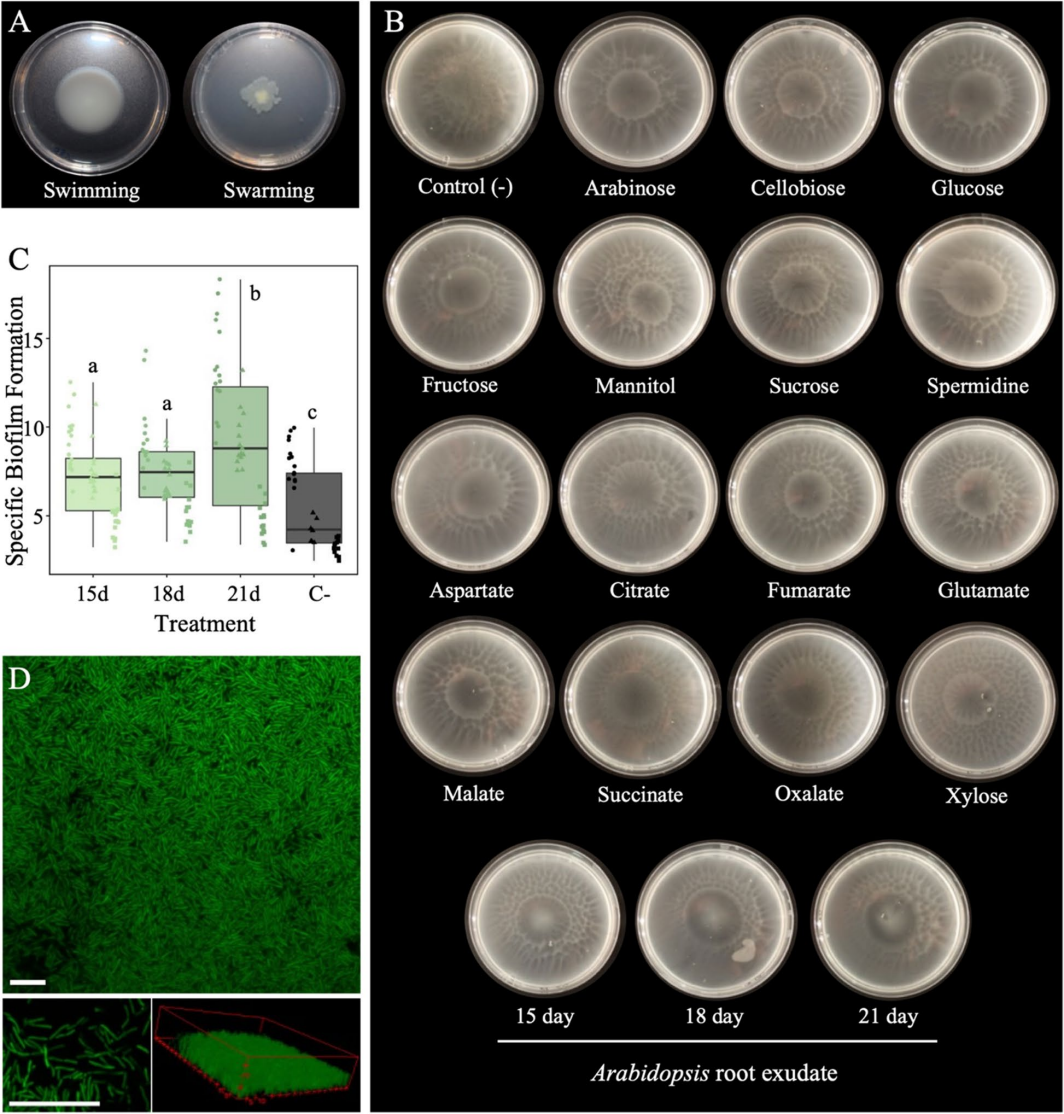
Motility, chemotaxis and biofilm-responses of *P. granadensis *CT364. **A** Swimming and swarming responses of *P. granadensis *CT364. Cultures were inoculated centrally into plates containing 0.3% (swimming) or 0.5% agar (swarming) and plates were incubated for 48 h. **B** Chemotactic response of *P. granadensis* CT364 towards to organic acids and carbon sources common in plant root exudates, as well as to Arabidopsis root exudates, was assessed. Chemotaxis buffer was used as a negative control (Control -). Chemotaxis responses were characterised as positive (+) when bacteria formed a halo around the application site or strong (+ +) when bacteria reached the application site after 30 min of incubation. Each plate is a representative of three replicates. **C** Biofilm formation in the presence of root exudates collected from 15-, 18- and 21-day-old Arabidopsis plants. Specific Biofilm Formation (SBF) was calculated by crystal violet staining following 4 h incubation. The assay was conducted with three biological replicates, represented by different point shapes. The statistically significant differences are represented by lettering. **D** CLMS micrographs of Z-stack analysis of *P. granadensis* CT364 fluorescence (CT364-pSNW2) biofilm formed on glass coverslip under stationary conditions. Scale bars = 10 μm

The genome of *P. granadensis* CT364 encodes the complete set of genes required for motility and chemotaxis, including those for flagella synthesis, type VI pili, chemotactic response proteins, methyl-accepting proteins (MCPs) and methyl-accepting chemotaxis sensors/transducer proteins (Table S1) [26, 170, 171]. Notably, *P. granadensis* CT364 harbours 38 MCP chemoreceptors, substantially higher than the bacterial average of 14 [89, 102]. Given that a high number of MCP chemoreceptors is often indicative of a plant-associated lifestyle [102, 118], the degree of plant specificity (DPS) of the MCP ligand-binding domains (LBD) was calculated [139]. The Analysis revealed that 50% of these MCP LBD sequences were ecologically annotated as belonging to plant-associated niches, including terms such as “Plants”, “Rhizoplane”, and “Arabidopsis rhizosphere” (Table S2). Furthermore, the low degree of similarity between *P. granadensis* CT364s’ MCP sequences and those in existing genomic catalogues underscores their uniqueness to the CT364 strain, and their potential functional role in plant–microbe associations. To validate these genomic predictions, we confirmed the motile nature of *P. granadensis* CT364 using swimming and swarming assays (Fig. 1A). We also assessed its chemosensory capabilities in response to compounds found within plant root exudates. *P. granadensis* CT364 exhibited positive chemotaxis towards both organic acids and other carbon sources typically found in Arabidopsis root exudates (Fig. 1B). Moreover, chemotactic responses were stronger when exposed to root exudates from 15-, 18-, and 21-day-old Arabidopsis plants compared to responses elicited by individual compounds. This enhanced response was characterised by bacterial accumulation directly at the exudate application site, as opposed to the halo formation observed with individual compounds. These findings demonstrate that CT364 not only possesses the genetic toolkit for effective chemotactic behaviour but that this translates into a measurable response that could facilitate its association with plant roots.

#### Biofilm formation

To investigate how plant-derived signals influence bacterial behaviour, biofilm formation in response to root exudates was assessed. Biofilm accumulation was notably enhanced in the presence of Arabidopsis root exudates, with the strongest response observed using exudates from 21-day-old plants (Fig. 1C). In parallel, *P. granadensis* CT364 engineered to express the reporter eGFP were seen to adhere to surfaces via dense biofilm formation and bacterial aggregation (Fig. 1D). At biofilm periphery, where fewer bacteria were attached, filamentation and semi-septated elongated cell clusters were observed. These variations likely reflect distinct stages of biofilm maturation and bacterial adaptation at different microenvironments.

During biofilm formation, cell imbibition is mediated by the secretion of extracellular matrix components, such as exopolysaccharides and adhesive proteins, which are often host-specific [24, 94]. Analysis of the *P. granadensis* CT364 genome shows that it may encode biosynthetic genes for the exopolysaccharides poly-N-acetyl-glucosamine (PGA; *pgaABCD*) [74, 94] and alginate (*algPQR algB kinB algFJLXGEK alg44 alg8*, *algD*) [23] (Table S1). In addition, the genome harbours genes that may encode adhesive proteins, such as non-fimbrial RTX proteins and ‘adhesin involved in diffuse adherence’ (*aidA*). Genes for fimbrial structures were also identified, such as Type IVa pili (*fimUTV*), tight adherence (Tad) pilus (including the fimbrial low-molecular weight protein *flp*; and *cpaABCEF* and *tadBCDG* operons) [160], as well as amyloid fimbrils of the *fap* system (Table S1). Additionally, sequences encoding the LapA surface protein and its associated transport proteins *(lapCDE)* were also present (Table S1). LapA is recognised as a crucial component in the early stages of plant surface attachment, biofilm formation and overall bacterial fitness in the rhizosphere [99, 122]. Together, these findings indicate that *P. granadensis* CT364 is well-equipped both genetically and phenotypically for robust biofilm formation. The effective secretion of extracellular matrix components and multiple adhesion factors likely enhances its ability to colonise plant surfaces and thrive in the competitive rhizosphere, supporting the hypothesis that some of its life is spent as a plant endophyte.

#### Rhizosphere and endophytic colonisation

**Fig. 2:**
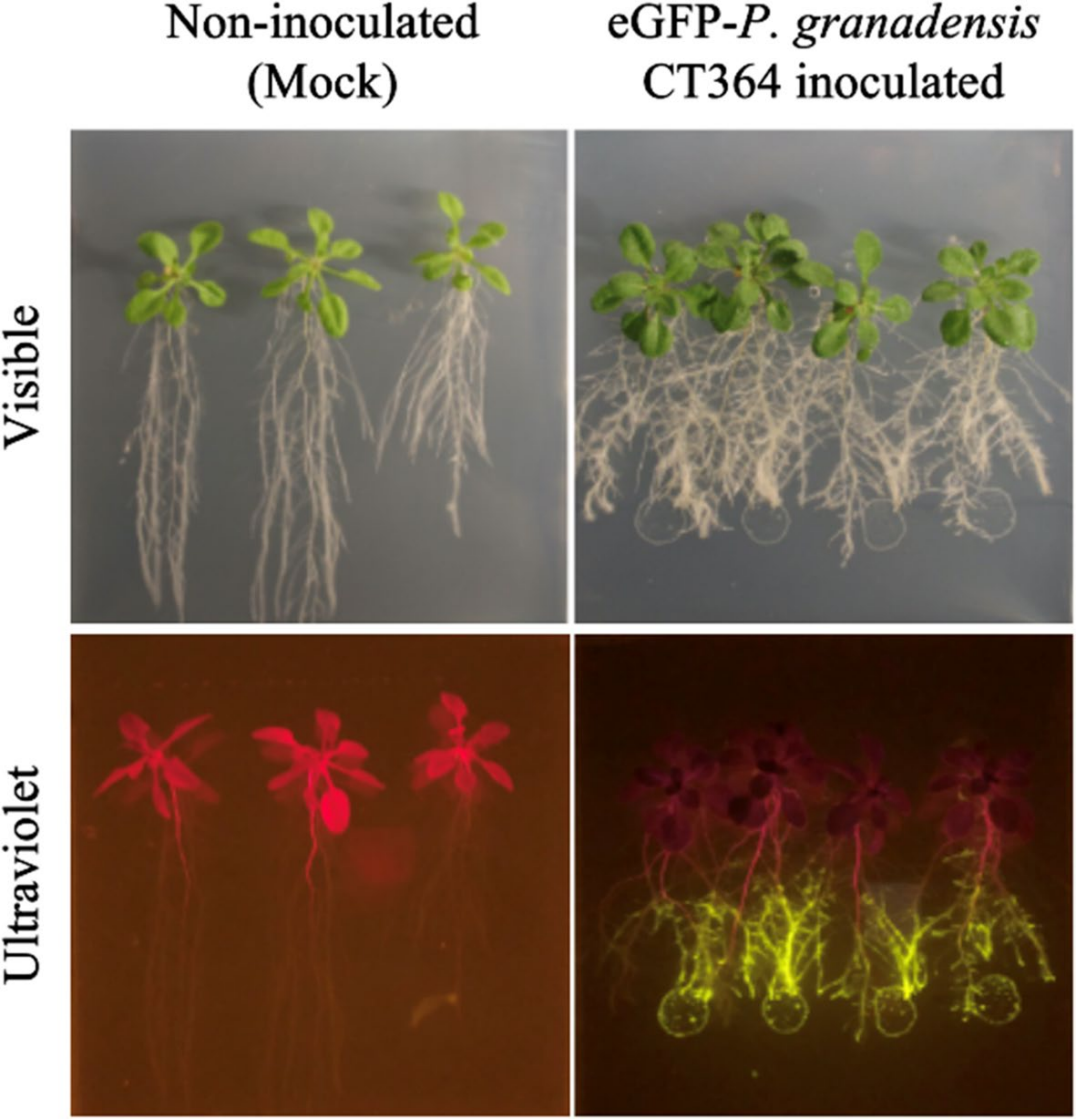
*P. granadensis* CT364 colonisation of Arabidopsis. Seven-day-old Arabidopsis seedlings were transferred to 1/2 MS 3% sucrose agar plates, and inoculated with 50 ul drops of GFP-expressing CT364 culture 5 cm away from the seedlings. The assay was conducted with 4—5 plants per plate and three plates per treatment. After two weeks incubation, plates were photographed under visible and ultraviolet light. The experiment was replicated three times (*n* = 3)

**Fig. 3:**
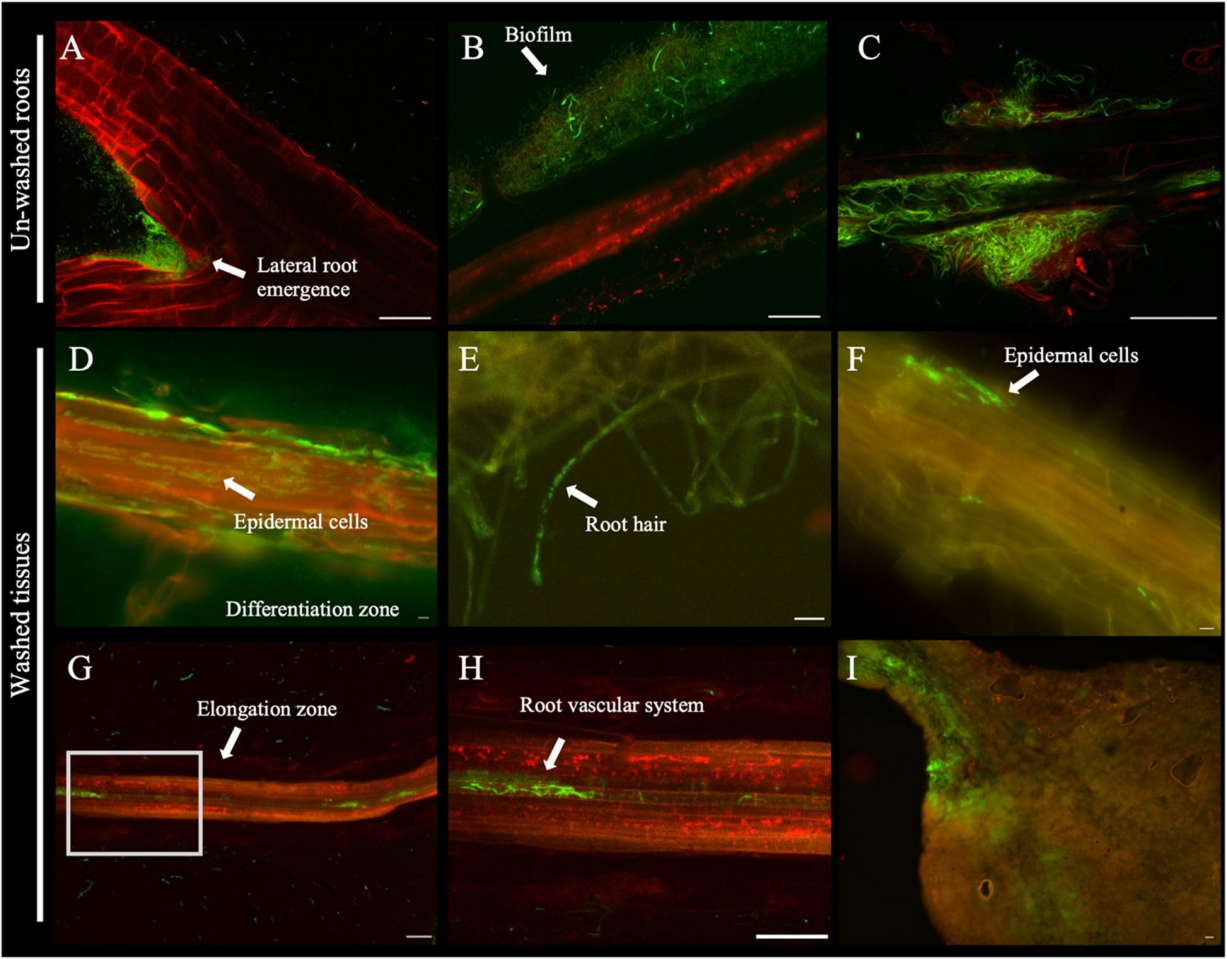
Visualisation of *P. granadensis* CT364 expressing eGFP following two weeks co-cultivation with Arabidopsis. Seedlings were vertically grown on ½ MS 1% sucrose agar plates. Following sowing, seedlings were inoculated with a suspension of eGFP-expressing *P. granadensis* CT364 cells. The presence of *P. granadensis* CT364 was visualised using confocal microscopy with two channels emitting light at different wavelengths (488 and 568 nm). Externally, bacterial attachment to the root surface was visualised for both unwashed (**A**-**C**) and washed (**D**) plant roots. Internally, *P. granadensis* CT364 was observed within root hairs (**E**) epidermal cells (**F**), in the root vascular systems (**G**-**H**) and in leaves (**I**). The scale bar represents 40 μm in all panels. Arrows indicate specific plant zones

To assess the colonisation dynamics of *P. granadensis*, we tested its ability to interact with Arabidopsis roots without direct contact. In-distance inoculation with the *P. granadensis* CT364 strain expressing eGFP demonstrated that it can colonise the Arabidopsis rhizosphere in a contact-independent manner (Fig. 2). To determine whether *P. granadensis* CT364 is an endophytic strain, Arabidopsis seedlings grown vertically on ½ MS media plus 1% sucrose, were co-cultivated with eGFP-expressing *P. granadensis*. This allowed documentation of root colonisation under controlled conditions on three-week-old seedlings. The assay visualised *P. granadensis* using both washed and unwashed Arabidopsis plants allowing visualisation of microbial adhesion to the rhizoplane (unwashed) and colonisation of the internal tissue (washed). Both washed and unwashed plants developed biofilm structures coating the rhizoplane (Fig. 3A-D). Unwashed plants exhibited more extensive cellular accumulation and filamentation, whereas washed plants showed a reduction in these features. Bacterial distribution on the surface was uneven, with prominent accumulation of bacteria on the lateral roots and in the elongation and differentiation zones. Following epidermal cell attachment, washed plants revealed internal colonisation of root hairs (Fig. 3E) and intracellular spaces (Fig. 3F). In addition, *P. granadensis* CT364 was observed moving into the vascular system (Fig. 3G-H, Supplementary Video) and disseminating systemically into aerial tissues such as shoots and leaves (Fig. 3I). Notably, while colonisation of root hairs and intracellular spaces appeared as aggregated colonies, *P. granadensis* CT364 in vascular systems showed as discontinuous bacterial filaments. Overall, these results confirm that *P. granadensis* CT364 can effectively colonise Arabidopsis by first establishing robust rhizoplane biofilms and subsequently penetrating internal root tissues prior to moving into vascular structures. This colonisation capability further underscores its role as a plant endophyte with potentially systemic distribution.

### * P. granadensis* CT364 is genomically similar to other beneficial PGP Pseudomonas species

**Fig. 4:**
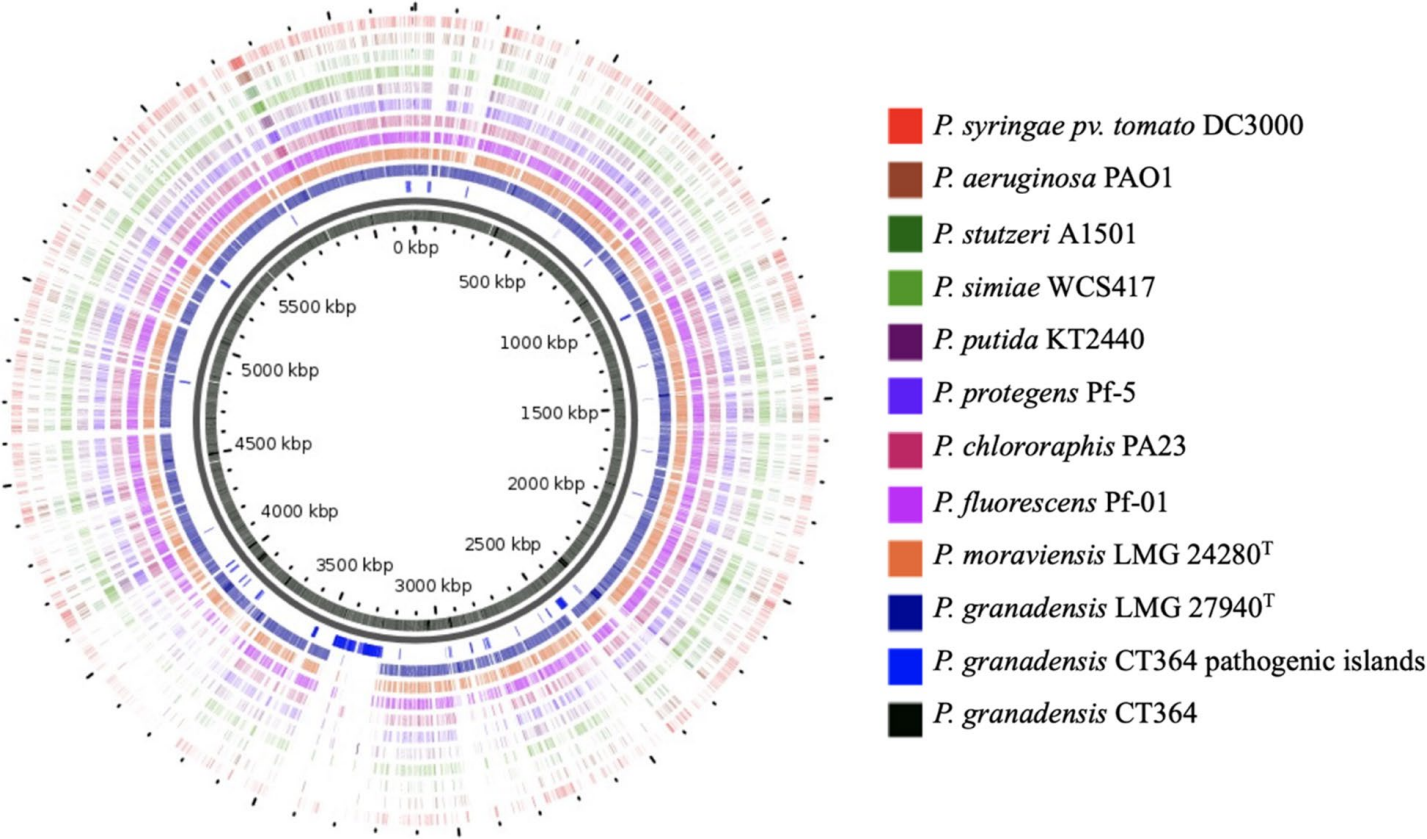
Genomic similarity of *P. granadensis* CT364 to other pseudomonads. A Blast atlas comparison between the *P. granadensis* CT364 genome (black) and pathogenicity islands (dark blue) and 11 beneficial and non-beneficial Pseudomonas strains. The absence of colour signifies the absence of gene homology compared to the query genome. The diagram was generated by the Gview server

To genomically assess its potential role as a PGPB as well as investigating its pathogenic potential, we performed comparative genomic analyses against both beneficial and pathogenic *Pseudomonas* species. As anticipated, *P. granadensis* CT364 showed high genomic homology, similar gene arrangement and a large set of shared orthologous genes with the type strain of *P. granadensis* (LMG 27940^ T^) as well as with the model PGP strain *P. fluorescens* Pf-01 (Fig. 4). Its similarity to the PGP strains *P. moraviensis* LMG 24280^ T^*, P. putida* KT2440*, P. simiae* WCS417 and *P. stutzeri* A1501 was lower. The two pathogenic strains (*P. aeruginosa* PAO1 and *P. syringae pv. tomato* DC3000) demonstrated the least homology to *P. granadensis* CT364. Pan-genomic analyses also confirmed that *P. granadensis* CT364 shares greater similarity with beneficial PGP species compared to pathogenic species (Table S3). In particular, *P. aeruginosa* PAO1 was distinguished from the PGP pseudomonads by a high number of exclusively absent genes common to beneficial *Pseudomonas* species, and by having the lowest number of accessory genes in this data set (Table S3).

Analysis of CT364’s pathogenicity islands provided further support for a beneficial role in plant–microbe interactions. Alignment of pathogenicity islands in *P. granadensis* CT364 with genomes of other pseudomonads was examined (Table S1). This analysis revealed limited alignment, including with the *P. granadensis* LMG 27940^ T^ strain from the same species, highlighting the unique composition of CT364’s pathogenicity islands. The genomic content of *P. granadensis* CT364 pathogenicity islands was examined. The genome lacks genes commonly associated with plant pathogenic *Pseudomonas* species, such as pathogenic toxins and the type III secretion systems [31, 61]. Instead, it possesses genes associated with osmoprotection, the type VI secretion system (T6SS), siderophore production, biofilm formation and insecticidal protein synthesis (Table S1) [51, 137, 144, 186]. The type VI secretion system (T6SS) structural and regulatory genes (*tssABCEFGHIJKLM, tagH, ppkA, pppA*) were found in a single putative operon, and T6SS-related effectors (*hcp*, *vgrG*, PAAR domain-containing protein and Rhs effector proteins) were dispersed throughout the genome (Table S1), as is common in plant-associated bacteria [21]. In summary, comparative genomic analysis positions *P. granadensis* CT364 within the beneficial PGP group. The absence of key pathogenicity determinants combined with the presence of mechanisms associated with plant growth promotion and stress adaptation underscores its potential as an agriculturally relevant, beneficial, plant-associated bacterium.

### *P. granadensis* CT364 enhances Arabidopsis growth under both control and saline stress conditions

Analysis of the *P. granadensis* CT364 genome revealed a high abundance of osmoregulatory genes (Table S1). These include genes involved in potassium uptake (*trkAH, kup, kefB, kdpABCDE*) [132], choline uptake (*choVXW*, [3]), and the synthesis of compatible solutes such as proline and glycine betaine (*prop, betAB, betI, betTUX, gbcAB, dgcAb*) [30, 50, 96, 109] (Table S1). In addition, the *P. granadensis* CT364 genome contains transport and catabolic genes for gamma-aminobutyrate (GABA) (*gabP, gabD-gabP*), a plant-produced regulator of growth and development under stress conditions (Table S1) as well as genes for the biosynthesis of osmoprotectants such as spermidine (*aguAB, speA, speD, speE, osmVWXY, potABCD, potEFGHI*) [93] and trehalose (*treS, malZ, malQ, treYZ, glgB, treC, treRP*) [173]. These results suggest *P. granadensis* CT364 has the capabilities to mitigate plant stress through the synthesis of osmoprotectants and suppression of GABA-induced stress responses when plants are exposed to abiotic and biotic stresses [92, 140].

To validate these genomic findings, we assessed the strain’s resistance to osmotic stress in liquid cultures by exposing it to increasing concentrations of NaCl and polyethylene glycol (PEG), which simulate salinity and drought conditions, respectively (Figure S2). Remarkably, the strain survived up to an osmotic pressure of ΔΨo = −2.5 MPa, equivalent to media supplementation of 3.2% NaCl and 40% PEG [68, 70, 175]. Notably, growth was more adversely affected by PEG-induced osmotic pressures, starting at a water potential of ΔΨo = −0.5 MPa, compared to equivalent water potentials induced by NaCl. These results, combined with those describing its ability to colonise plant tissues and the previous reports of screens for plant growth-promoting activities [110], lead to the hypothesis that *P. granadensis* CT364 may enhance plant performance under osmotic stress.

**Fig. 5:**
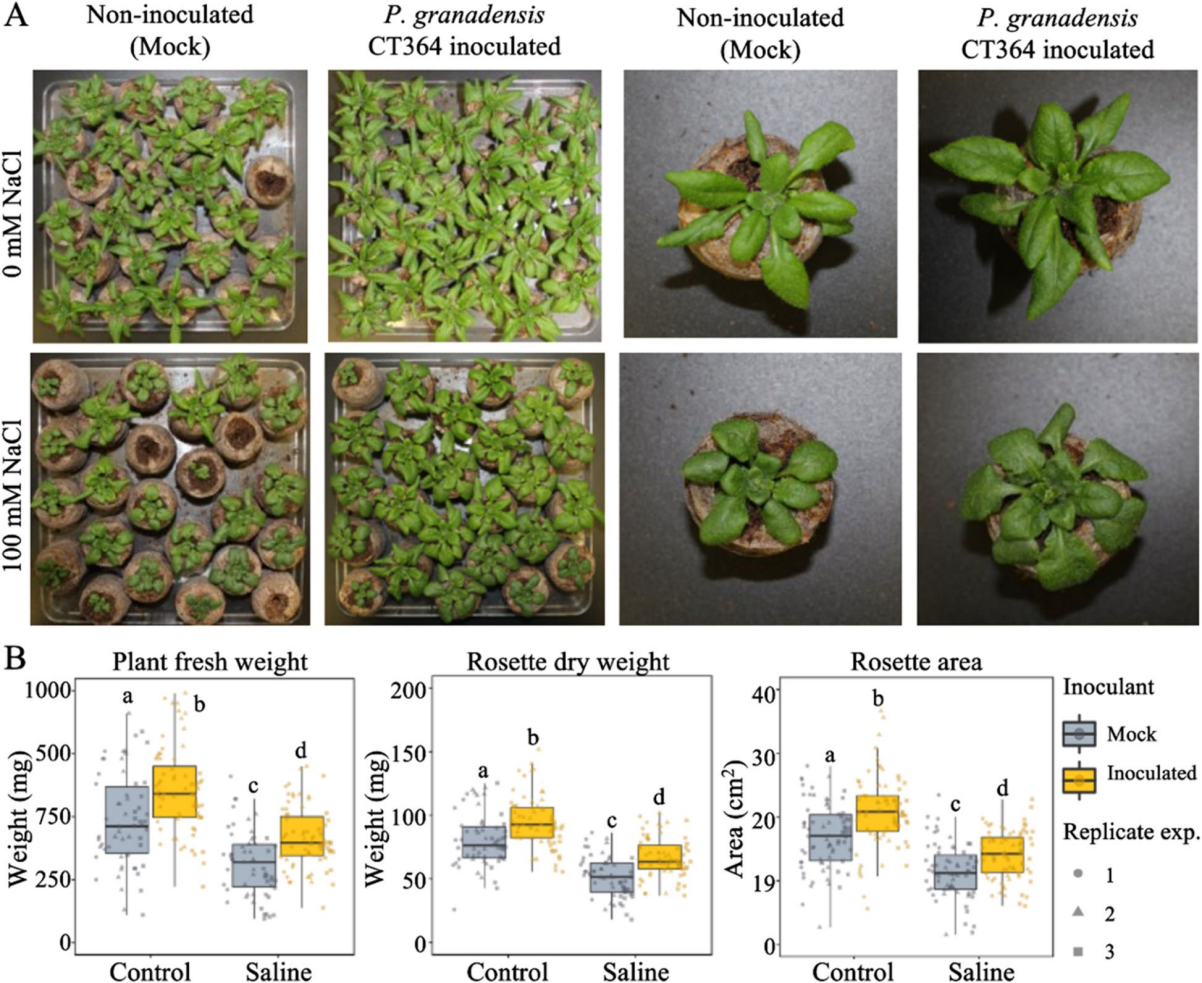
Arabidopsis growth following inoculation with *P. granadensi*s CT364. Plants were sown in ½ MS agar plates for a week before seedlings were transferred to Jiffy pellets and inoculated with either MgSO4 (Mock) or *P. granadensis* CT364 suspension. Plants were grown for a total of 28 days with watering every three days. Salinity was simulated by single irrigation with a 100 mM NaCl solution one week after inoculation. Plant growth promotion was evaluated via visual examination (**A**) and measurement of plant fresh weight, rosette dry weight and rosette area (**B**). The mean and standard deviations of three replicates with 25 plants per treatment are displayed in box plots. Shapes represent three different replicate experiments. The statistical significance of the results was calculated using two-way ANOVA and Tukey's text (*p*-value < 0.05) with letters indicating significantly different means

**Fig. 6:**
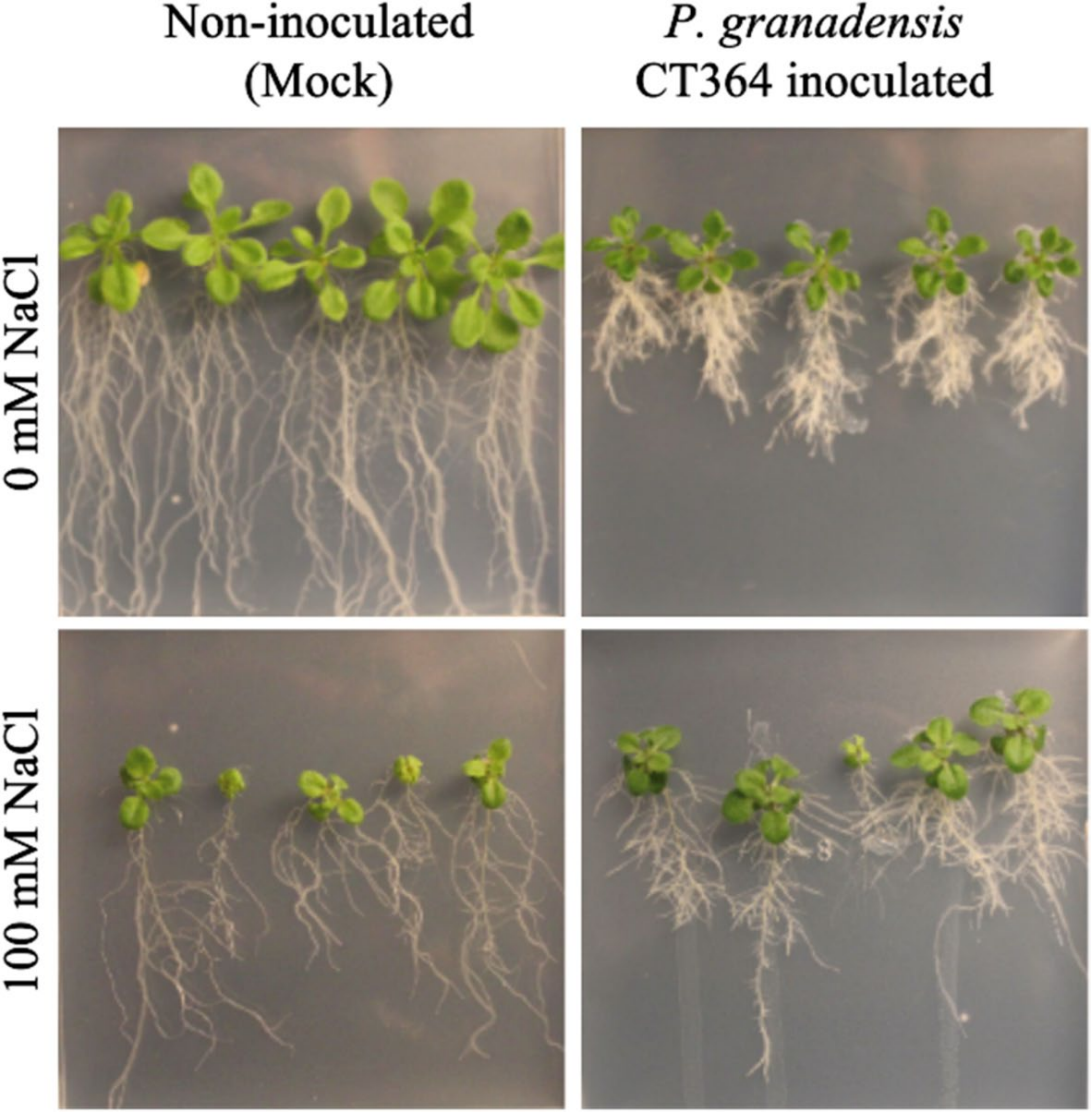
Root architecture of treatment. Arabidopsis following inoculation with *P. granadensis *CT364. Seven-day old Arabidopsis seedlings were inoculated with a suspension of *P. granadensis* CT364 in 10 mM MgSO_4_ or in a 10 mM MgSO_4_ solution (mock). Following inoculation seedlings were transferred to fresh ½ MS 1% sucrose agar plates, with or without 100 mM NaCl. Plates were incubated vertically, and images were taken 14 days post-inoculation. Representative results shown. The assay was performed in triplicate, with five plants per plate and five plates per treatment

Salt stress is frequently employed to evaluate the efficacy of PGPB because it combines osmotic stress with ion toxicity, permits uniform application, and overlaps with plant responses to other abiotic stressors, such as cold temperature and drought [13, 155, 188]. Accordingly, the beneficial effects of *P. granadensis* CT364 for plant growth were evaluated under soil-mimicking axenic and in vitro conditions using Arabidopsis. Plant growth promotion was assessed 28 days post seed germination by measuring whole plant fresh weight, rosette dry weight, and rosette area. Two-way ANOVA revealed a significant increase in all parameters in plants inoculated with *P. granadensis* CT364 compared to controls (Fig. 5). These outcomes were maintained under both control and saline conditions (Fig. 5). Specifically, whole plant fresh weight increased by 24% under control conditions and 33% under salinity stress (Table S4), while rosette dry weight increased by 17.5% and 29.8%, respectively. Salt stress reduced the rosette area of non-inoculated plants by 32%, while inoculation with *P. granadensis* CT364 resulted in an approximate 23% increase in rosette area under both control and stress conditions. Regarding leaf morphology, exposure to osmotic stress had an impact on both the shape and size of the leaves. Changes in leaf shape induced by salinity however, appeared independent of bacterial treatment. Finally, in vitro assays on agar plates containing 100 mM NaCl corroborated these findings. Three-week-old *P. granadensis* CT364-treated seedlings grown under salt stress exhibited increased rosette biomass along with significant modifications to root architecture, including reduced primary root length, but enhanced formation of lateral roots and root hairs compared to mock inoculated plants (Fig. 6).

Complementing these observations on changes to root architecture, we examined the potential for *P. granadensis* CT364 to produce indole-3-acetic acid (IAA). IAA is a key phytohormone that regulates various aspects of root development and overall plant growth, including lateral root initiation and root hair formation, resulting in a plant’s ability to increase access to water and nutrients. We verified the presence of IAA biosynthetic genes in *P. granadensis* CT364 via the indole-3-acetamide pathway (*trpABCDE*, *iaaM*, and *iaaH*) [183]. Consistent with this, *P. granadensis* CT364-inoculated seedlings displayed pronounced IAA-mediated effects, such as stimulated root growth, increased lateral branching, and a higher density of root hairs (Fig. 6). The altered root architecture likely increases water and nutrient uptake and enlarges the surface area for beneficial interactions with soil microorganisms, potentially contributing further to the plant’s resilience under osmotic stress. Collectively, these findings highlight the multifaceted role of *P. granadensis* CT364 in promoting plant growth. Its robust osmoregulatory capacity, suggesting a multifaceted strategy by which *P. granadensis* CT364 may mitigate abiotic stress, coupled with the ability to modulate root architecture through IAA production, supports its potential role as a PGPB capable of mitigating the adverse effects of abiotic stress and enhancing overall plant performance.

### Biomineralization capabilities of *P. granadensis* CT364

*P. granadensis* CT364 has been demonstrated to solubilise phosphate [110]. Here, genomic analysis revealed a full complement of genes involved in phosphate metabolism. These include genes encoding the enzymes responsible for dephosphorylation and transport of organic compounds, such as phosphatases (*phoX*, *phoB*), kinases (*ppk*, *phnA*, *phnB*) and C-P phosphonate lyases, alongside high-affinity phosphate and phosphonate transporters. Regulatory components, including the phosphate sensor PhoR and regulatory proteins (*phoB* and *phoU*) [76, 142] (Table S1) were also present. Additionally, the *P. granadensis* CT364 genome contains four genes encoding phosphate-binding DING proteins (*ptsS*, *pstA*, *pstB* and *pstC*) which have been implicated in biomineralization. Other genes for production and secretion of organic acids (citrate, malate, malonate, gluconate, 2-ketogluconate, and lactate) into the soil for mineral phosphate solubilisation were also found in the *P. granadensis* CT364 genome [172]. Together, this analysis identifies the genetic components likely to underpin the previous finding that *P. granadensis* CT364 possesses an efficient phosphate uptake system.

*P. granadensis* CT364 has also been shown to produce siderophores [110]. Here, genome analysis shows that its genome harbours a well-defined pyoverdine biosynthetic cluster (pvdAE, pvdIJ, pvdLMNOPQ, FpvR, pvdS, pvdYZ) essential for siderophore production. Along with 26 TonB-dependent receptors that facilitate iron uptake, multiple regulatory proteins (including Fur, GacS/GacA, RpoS, and quorum sensing autoinducers) were identified [43, 133] (Table S1). Taken together this indicates that *P. granadensis* CT364 possesses an efficient iron-acquisition system.

### Biocontrol functions

The Virulence Factor Database [95] was used for the in silico identification of genes with biocontrol functions. This approach enabled the detection of several biosynthetic gene clusters, including those responsible for the synthesis of hydrogen cyanide (*hcnABC*) [90], phenazine production (*pheZ*) [39, 77], and the presence of pyocin domain-containing proteins [115]. These latter are antibacterial proteins that target and disrupt cellular functions of competing closely related bacteria, and whose expression is linked to stress exposure, potentially providing a competitive advantage over them in the same ecological niche. However, functional analysis of pyocins outside *P. aeruginosa* is limited. In addition, eight putative insecticidal proteins were identified further suggesting a multifaceted role in host protection (Table S1). Seven of them were annotated to be Tc toxins, known for high activity against insects [108, 181]. Functional validation supported these genomic predictions. A colorimetric picrate assay confirmed hydrogen cyanide production by CT364 (Figure S3). Antimicrobial activity was assessed using *Bacillus subtilis* reporter strains. Results indicate that *P. granadensis* CT364 induced a response in the *fabHA* reporter, associated with fatty acid biosynthesis inhibition (Figure S4), and produced a weak inhibition zone (~ 1 mm; Table S5). These results suggest limited antibacterial activity under the tested conditions but indicate the presence of a bioactive compound affecting lipid metabolism in *B. subtilis*.

### Metabolic capabilities and detoxification mechanisms of *P. granadensis* CT364

The *P. granadensis* CT364 genome comprises 277 transporter genes for nutrient uptake, along with genes involved in the biosynthesis and degradation of polyhydroxyalkanoates, which serve as carbon storage molecules [126, 149] (Table S1). The genome displays considerable metabolic versatility for the utilisation of plant-derived substrates. In particular, its genome contains genes for the assimilation of plant cell wall monomers, such as xylose (*xylABFGHR*), and for the breakdown of structural components from bacterial and fungal cell walls, including N-acetylglucosamine (N-GlcNAc) [20].

In addition to nutrient acquisition, *P. granadensis* CT364 is equipped to manage and detoxify a range of toxic plant-derived compounds. The genome includes various detoxification enzymes that mitigate the effects of compounds such as reactive oxygen species, nitriles, cyanates and nitric oxide (Table S1) [60, 72, 88, 162]. Notably, *P. granadensis* CT364 harbours a plant-induced nitrilase gene together with a corresponding transcriptional regulator, as well as a large collection of efflux pumps that belong to the Resistance Nodulation cell Division (RND) and Major Superfamily types (Table S1), further emphasising its potential for adaptive responses to the rhizosphere and internal environments in the plant.

Beyond adaptations to organic compounds, *P. granadensis* CT364 possesses a comprehensive suite of genes to help it mitigate the negative effects of exposure to heavy metals and toxic compounds. Key resistance genes include those involved in chromium resistance (*chrAB, chrR*) [128], copper detoxification (including *copCDGZ, copAB*, multicopper oxidases, *cusSR, cueR, cutE, CorC*, and copper metallochaperones) [56, 123, 184]. Additional resistance genes were identified to metals such as cobalt, zinc, cadmium and arsenate (*arsRB, arsCH, arsC*) [180], as well as macrolide antibiotics (*macAB*) [64] and fluoroquinolones (DNA gyrase and topoisomerase, *gyrAB, parCE*) [185]. Complementary to these capabilities, the genome possesses a wealth of other detoxification mechanisms, including efflux systems, cytosolic sequestration mechanisms, and enzymatic detoxification pathways (Table S1) [56, 103].

Experimental validation supported these genomic insights. *P. granadensis* CT364 demonstrated remarkable tolerance to heavy metals, maintaining growth in the presence of lead and cadmium at concentrations up to 1000 mg/l (corresponding to 8.89 mM and 4.8 mM, respectively (Figure S5). A bibliographical study compiling data on heavy metal-resistant, plant-associated bacteria—expressed in terms of minimum inhibitory concentration (MIC)—highlighted *Pseudomonas* as one of the most resistant genera to cadmium and lead, following *Klebsiella* and *Enterobacter* [63]. The study reported average MIC values for cadmium and lead below 5 mM among *Pseudomonas* PGPB strains, underscoring the high resistance of CT364. The strain also demonstrated growth in challenging concentrations of zinc at 7.64 mM (500 mg/l), copper at 7.86 mM (500 mg/l), chromium at 1.92 mM (100 mg/l) and cobalt at 0.85 mM (50 mg/l). For comparison, a study isolating heavy metal-resistant PGPB from a coal dumping site reported MIC ranges of 5–9 mM for copper, 5–8 mM for zinc, 0.6–3 mM for lead, 0.5–8 mM for chromium, and 0.5–3 mM for cadmium [19]. The present study demonstrates the significant heavy metal tolerance exhibited by CT364 reinforcing its potential application in phytoremediation and plant growth promotion in heavy metal-contaminated soils.

Overall, the metabolic diversity and extensive detoxification systems encoded within the *P. granadensis* CT364 genome highlight its adaptability to challenging environments. These not only enhance the survival and competitiveness of *P. granadensis* CT364 in diverse ecological niches, but also position it as a promising candidate for biotechnological applications.

## Discussion

We characterised the endophytic nature and PGP abilities of *P. granadensis* CT364 using a combination of genomic analysis and experimental validation. The strain exhibited traits prerequisite for endophytic colonisation confirmed by its motility, responsiveness to plant compounds, and capacity for biofilm formation. These characteristics enable *P. granadensis* CT364 to colonise Arabidopsis roots in a contact-independent manner. Biofilms observed on both washed and unwashed roots are in line with findings from other endophytes such as *Gluconacetobacter diazotrophicus*, *Bacillus cereus*, *P. fluorescens* and *P. simiae* PICF7 in that biofilm formation is required prior to colonisation [75, 105, 111, 179]. Similar associations between biofilm formation and PGP phenotypes have been reported for *B. subtillis, P. fluorescens, P. putida* and *P. chlororaphis*, [38, 60, 71]. The presence of *P. granadensis* CT364 inside root hairs further suggests this as a point of entry, as has been reported for other olive tree endophytes, *P. putida* PICP2 *and P. fluorescens* PICF7 [127].

Genome Annotation and in silico predictions identified 564 genes associated with rhizosphere competence and PGP traits [67, 101]. These genes suggest the potential molecular mechanisms underpinning nutrient uptake, stress alleviation, and developmental modulation of plants. Additionally, analyses of MCPs identified chemoreceptors critical for plant–microbe interactions and niche specialisation [139, 161]. The PGP effects of *P. granadensis* CT364 were validated using Arabidopsis. Inoculation of plants with this strain mitigated the significant 35% reduction in biomass seen under saline conditions where plants were not inoculated. This observation, together with previous reports of PGP and rooting induction in olive, rapeseed and other plant species [110], indicates a broad host range for this bacterium. By contrast, *P. fluorescens* PICF7, an endophyte of olive trees and a potent growth promoter of barley (*Hordeum vulgare L*.), Arabidopsis and banana (*Musa acuminata L.*) [17, 32, 97, 106, 111] has only been described as an endophyte of olive trees. The combined PGP effect and endophytic colonisation of the model plant Arabidopsis under controlled laboratory conditions therefore make *P. granadensis* CT364 an excellent model for studying PGP endophytes.

Growth promotion may be mediated in a number of different ways. In vitro, *P. granadensis* CT364 enhanced root formation, likely due to its production of the auxin IAA [110]. Other PGPB, such as *Bacillus velezensis* FZB42 and *P. fluorescens* WCS374, have also been demonstrated to induce lateral root and root hair formation in Arabidopsis through the secretion of auxins [2, 164]. It is hypothesised that increased lateral root ramification and root hair formation expands the root surface area, enabling plants to better sustain growth under conditions of nutrient deficiency or other abiotic stresses [86, 100, 135]. In addition to changes in plant morphology, *P. granadensis* CT364 also exhibits traits beneficial for nutrient uptake and stress alleviation. It produces siderophores and solubilises phosphate, facilitating nutrient uptake by plants [110]. Genome analysis revealed the presence of genes for spermidine and trehalose biosynthesis, as well as catabolism of GABA, consistent with a potential for osmotic stress alleviation. Spermidine is known to enhance plant tolerance to salinity, temperature, oxidative, and drought stress [187] by promoting root system development, boosting antioxidant activity, reducing plant ethylene levels, and increasing abscisic acid content [165, 168, 178]. Trehalose alleviates plant abiotic stress by stabilising essential biomolecules, modulating antioxidant activities, and regulating plant stress-related genes [134, 154, 167]. Conversely, GABA is produced and accumulated by plants in response to abiotic and biotic stress and plays a role in regulating growth and development upon biotic and environmental stress exposure, as well as recruiting microbial communities [47, 140, 150, 163, 174]. The presence of GABA catabolic genes in the *P. granadensis* CT364 genome indicates its potential ability to degrade and utilise plant-produced GABA, alleviating GABA-induced stress response in plants [92, 140].

Comparative genomics demonstrated high conservation and orthology with the model PGP *P. fluorescens* Pf-01, while clearly differentiating CT364 from pathogenic *Pseudomonas* strains. Whole genome comparisons revealed the presence of unique pathogenicity islands in *P. granadensis* CT364. These pathogenicity islands contain genes involved in bacterial environmental adaptation. We hypothesise that horizontal gene transfer played a significant role in the genomic differences observed when comparing this strain to its closest relative *P. granadensis* LMG 27940^ T^. This is a common phenomenon in other soil bacteria, facilitating the acquisition of traits for colonising the plant rhizosphere and endosphere [69, 78]. Notably, *P. granadensis* CT364 also harbours biotechnologically relevant features such as the type VI secretion system, insecticidal toxins, and the potential to synthesise biocontrol molecules. The endophytic nature of the strain renders its T6SS a valuable tool for agricultural biotechnology applications [28] while the Tc toxins have the potential to serve as substitutes for *Bacillus thuringiensis* toxins [108, 181].

Finally, *P. granadensis* CT364 was isolated from the arid region of the Iberian Pyrite Belt, known for its abundant pyrite and sulphide deposits of copper, zinc and lead [130, 141]. This harsh habitat has likely driven the evolution of robust stress response mechanisms in this strain. Consistent with this environmental origin, the strain exhibits high tolerance to salinity, osmotic stress, and heavy metal contamination. Genome analysis revealed numerous genes associated with the degradation of toxic compounds from both plant and microbial sources, potentially enhancing its carbon source utilisation and maximising survival in the rhizosphere [29, 114, 157]. The presence of plant-induced nitrilases in the strain genome suggesting that *P. granadensis* CT364 can sense plant signals and regulate the expression of detoxification genes accordingly. Furthermore, an elevated number of RND efflux pump gene families, which are known to play roles in antibiotic resistance and the initial stages of endophytic colonisation and survival in plant tissues in *P. putida* and *Erwinia chrysanthemi* were identified [18, 55]. The combination of phenotypic tolerance to abiotic stresses and a genomic repertoire rich in detoxification and efflux mechanisms underpins the ability of *P. grandensis* CT364 to survive in these adverse environmental conditions.

## Conclusions

A combination of genomic insights and experimental data supports the designation of *P. granadensis* CT364 as a PGP endophytic bacterium. Its ability to promote plant growth under both control and stress conditions, together with its genomic potential for environmental adaptation and biocontrol, makes it a promising candidate for agricultural and biotechnological applications and a new model system for exploring beneficial plant–microbe interactions. While validation in agronomically relevant crops and under field or semi-field conditions is essential for assessing practical applicability, multiple reports of positive interactions with diverse hosts (including olive, rapeseed, beans, and more recently, tomato) underscore its potential beyond the Arabidopsis model. At the same time, the Arabidopsis system described here provides a complementary platform for dissecting mechanisms of plant–microbe interaction under controlled conditions, thereby informing and guiding future translational studies.

## Supplementary Information

Supplementary Material 1.

Supplementary Material 2.

Supplementary Material 3.

## Data Availability

With the exception of the complete genome sequence of *P. granadensis* CT364, all data supporting the findings of this study are available in the Supplementary Information. The *P. granadensis* CT364 genome dataset was deposited into the NCBI database under accession number CP069352.1 and is available at the following URL: [https://www.ncbi.nlm.nih.gov/nuccore/CP069352.1] (https://www.ncbi.nlm.nih.gov/nuccore/CP069352.1). Raw data underpinning the tables and figures are available at 10.25405/data.ncl.30022390.
