# Supplementary material for: Genomic and functional analyses reveal Pseudomonas granadensis CT364 is a plant growth-promoting endophyte
Source: BMC Microbiol. 2025 Oct 10;25:651. doi: 10.1186/s12866-025-04308-6 (PMC12513076; doi:10.1186/s12866-025-04308-6)
Supplement: Supplementary file 1 — Supplementary Material 1. [file 12866_2025_4308_MOESM1_ESM.docx]

**
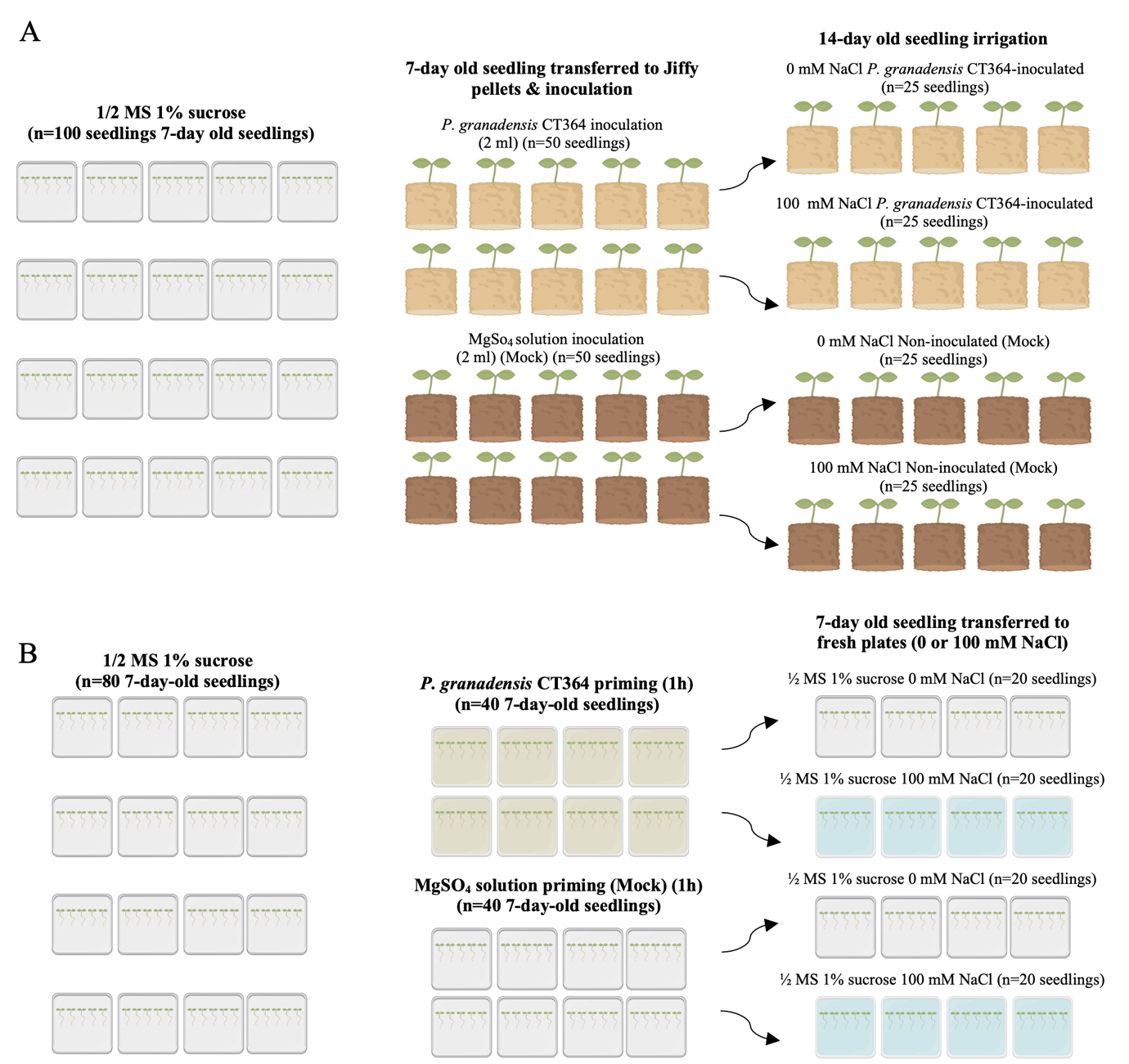
**

**Figure S1. Arabidopsis growth assays: experimental design. A) Soil-mimicking assays.** Seven-day-old Arabidopsis seedlings grown in ½ MS plates were transferred to Jiffy-7 41 mm peat pellets and inoculated with 2 mL *P. granadensis* CT364 bacterial solution (OD_600_ = 0.02) or 10 mM MgSO_4_ sterile solution (Mock). Plants were watered every three days with sterile deionised water. Salinity was simulated by single irrigation with a 100 mM NaCl solution one week after bacterial inoculation (14-day old plants). The assay (*n* = 25 replicates) was repeated three times giving a total of ~75 replicates across three experiments. **B) *In vitro* assays.** Seven-day-old seedlings were primed with *P. granadensis* CT364 inoculum (OD_600_ = 0.02) or 10 mM MgSO_4_ (Mock). After one hour, seedlings were transferred to fresh ½ MS 1% sucrose or ½ MS 1% sucrose supplemented with 100 mM NaCl Petri dishes. Three-week-old plants were photographed. The assay included *n* = 20 replicates and was repeated three times, providing ~60 replicates across three experiments. All experiments were conducted in a plant growth chamber at 22 °C, 70% humidity, 16/8h photoperiod and100 μmol m^-2^ s^-1^.

Figure S2. *P. granadensis* CT364 growth under saline and osmotic stress simulated conditions, by the addition of NaCl or PEG respectively. The strain was cultured in TSB with varying water potentials from 0 (C+) to -4 MPa. Non-inoculated TSB was used as a negative control (C-). Growth curves were obtained by cell turbidity monitorisation in a plate reader (λ=600 nm) for 60 hours. The experiment was performed in triplicates with error bars show the standard deviation.

Figure S3. HCN production assessed by colour change of pipric acid-containing filter paper from yellow (no production) to orange (medium production) or red-brown (high production). Cultures were incubated for 72 hours before the colour change in the filter paper, placed on the lid of the plates, was photographed. Plates coated with sterile water (H_2_O) or *E. coli* DH5α cultures were used as negative controls, and *P. fluorescens* ATCC 13525 was used as a positive control for HCN production.


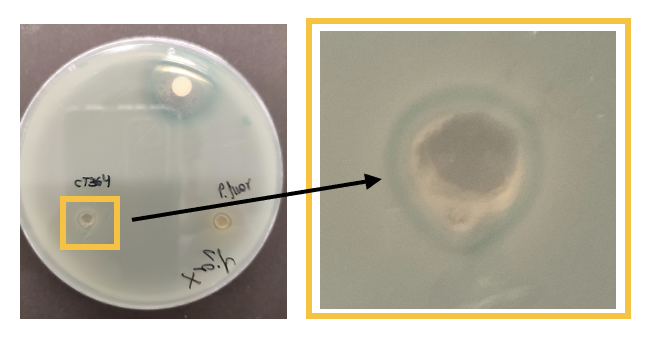


Figure S4. *P. granadensis* induced *Bacillus subtilis* LacZ-*yjaX* β-galactosidase activity (blue halo). Strains *P. granadensis* CT364 and *P. fluorescens* ATCC 13525 strains were tested for their antimicrobial mode of action after co-inoculation with *B. subtilis* reporter strains for 48 hours. A triclosan-impregnated paper strip was used as a positive control for *B. subtilis* *yjaX^ER^* growth inhibition and lacZ expression.

Figure S5. *P. granadensis* CT364 growth in different concentrations of heavy metals. Growth curves were obtained by optical density measurement in a plate reader (λ=600 nm) for 60 hours. To prevent visual confusion, not all growth curves generated at all concentrations are displayed. The diagrams display growth curves in non-supplemented TSB (C+), non-inoculated TSB (C-) or TSB supplemented with the MIC and other relevant concentrations. The colour gradient represents heavy metal concentration levels, with higher concentrations ranging from dark red (2500 µg/mL), red (1000 µg/mL), orange (500 µg/mL), light orange (250 µg/mL), to orange-white (100 µg/mL), and lower concentrations transitioning from blue-white (50 µg/mL), light blue (25 µg/mL), medium blue (5 µg/mL), to dark blue (0 µg/mL).The experiment was performed in triplicates, error bars show the standard deviation.
