# Supplementary material for: Genomic and functional analyses reveal Pseudomonas granadensis CT364 is a plant growth-promoting endophyte
Source: BMC Microbiol. 2025 Oct 10;25:651. doi: 10.1186/s12866-025-04308-6 (PMC12513076; doi:10.1186/s12866-025-04308-6)
Supplement: Supplementary file 2 — Supplementary Material 2. [file 12866_2025_4308_MOESM2_ESM.docx]

# **Supplementary Tables**

**Table S1. *P. granadensis* CT364 genes associated with plant growth promotion and endophytic lifestyle.**

| **Gene functional category** | | | | **Locus tag** | **Prokka/BLAST annotation; encoded protein** |
| --- | --- | --- | --- | --- | --- |
| **PLANT RHIZOSPHERE COLONISATION** | **Motility and chemotaxis** | **Flagellar motor proteins** | | JN757_03540 | chemotaxis protein/flagellar motor protein; MotA |
|  |  |  |  | JN757_08730 |  |
|  |  |  |  | JN757_03545 | chemotaxis protein/flagellar motor protein; MotB |
|  |  |  |  | JN757_08735 |  |
|  |  | **Flagellar biosynthesis** | | JN757_08440 | flagellar hook protein; FlgL |
|  |  |  |  | JN757_08435 | flagellar hook protein; FlgK |
|  |  |  |  | JN757_08430 | flagellar rod assembly protein; FlgJ |
|  |  |  |  | JN757_08425 | flagellar P-ring protein; FlgI |
|  |  |  |  | JN757_08420 | flagellar basal body L-ring protein; FlgH |
|  |  |  |  | JN757_08415 | flagellar basal body rod protein; FlgG |
|  |  |  |  | JN757_08410 | flagellar basal body rod protein; FlgF |
|  |  |  |  | JN757_21010 | Flagellar hook protein; FlgE |
|  |  |  |  | JN757_21015 | Flagellar basal-body rod modification protein; FlgD |
|  |  |  |  | JN757_21020 | Flagellar basal-body rod protein; FlgC |
|  |  |  |  | JN757_21025 | Flagellar basal-body rod protein; FlgB |
|  |  |  |  | JN757_21040 | Flagellar basal-body P-ring formation protein; FlgA |
|  |  |  |  | JN757_21045 | Negative regulator of flagellin synthesis (anti-sigma28); FlgM |
|  |  |  |  | JN757_21050 | Flagellar biosynthesis protein; FlgN |
|  |  |  |  | JN757_21055 | Flagellar brake protein (reponse regulator); YcgR |
|  |  | **Flagellar biosynthesis** | | JN757_08695 | flagellar biosynthesis regulator; FlhF |
|  |  |  |  | JN757_08670 | flagellar biosynthesis protein; FlhA |
|  |  |  |  | JN757_08665 | flagellar biosynthesis protein; FlhB |
|  |  |  |  | JN757_08660 | flagellar biosynthesis protein; FliR |
|  |  |  |  | JN757_08655 | flagellar biosynthesis protein; FliQ |
|  |  |  |  | JN757_08650 | flagellar biosynthesis protein; FliP |
|  |  | **Structural flagellar proteins** | | JN757_08645 | flagellar assembly protein; FliO |
|  |  |  |  | JN757_08640 | flagellar motor switch protein; FliN |
|  |  |  |  | JN757_08635 | flagellar motor switch protein; FliM |
|  |  |  |  | JN757_08630 | flagellar basal body protein; FliL |
|  |  |  |  | JN757_08625 | flagellar hook-length control protein; FliK |
|  |  |  |  | JN757_08605 | Flagellum-specific ATP synthase; FliJ |
|  |  |  |  | JN757_08595 | flagellar assembly protein; FliH |
|  |  |  |  | JN757_08590 | flagellar motor switch protein; FliG |
|  |  |  |  | JN757_08585 | flagellar M-ring protein; FliF |
|  |  |  |  | JN757_08580 | flagellar hook-basal body protein; FliE |
|  |  |  |  | JN757_08560 | flagellar assembly protein; FliT |
|  |  |  |  | JN757_08555 | flagellar biosynthesis protein; FliS |
|  |  |  |  | JN757_08550 | flagellar hook protein; FliD |
|  |  |  |  | JN757_08545 | flagellar protein; FlaG |
|  |  |  |  | JN757_08540 | Flagellin protein; FlaA |
|  |  |  |  | JN757_08770 | Uncharacterized homolog of the cytoplasmic domain of flagellar protein; FhlB |
|  |  | **Flagellar regulation** | | JN757_08565 | Flagellar regulatory protein; FleQ |
|  |  |  |  | JN757_08570 | Flagellar sensor histidine kinase; FleS |
|  |  |  |  | JN757_08575 | Flagellar two-component response regulator; FleR |
|  |  |  |  | JN757_08700 | Flagellar synthesis regulator; FleN |
|  |  | **Systems for signal transduction** | | JN757_06350 | two-component system, chemotaxis family, response regulator (EC:2.7.7.65); WspR |
|  |  |  |  | JN757_06330 | chemotaxis protein methyltransferase; WspC |
|  |  |  |  | JN757_06320 | methyl-accepting chemotaxis protein; WspA |
|  |  |  |  | JN757_06325 | chemotaxis-related protein; WspB |
|  |  |  |  | JN757_06335 | chemotaxis signal transduction protein; CheW |
|  |  |  |  | JN757_08720 | signal transduction histidine kinase; CheA |
|  |  |  |  | JN757_08720 |  |
|  |  |  |  | JN757_26350 |  |
|  |  |  |  | JN757_06345 | Chemotaxis response regulator protein-glutamate methylesterase; CheB |
|  |  |  |  | JN757_08725 |  |
|  |  |  |  | JN757_12780 |  |
|  |  |  |  | JN757_14025 |  |
|  |  |  |  | JN757_08715 | chemotaxis protein; CheZ |
|  |  |  |  | JN757_19605 | two-component system, chemotaxis family, response regulator; CheV |
|  |  |  |  | JN757_21035 |  |
|  |  |  |  | JN757_23075 |  |
|  |  |  |  | JN757_14020 | Multidomain signal transduction protein including CheB-like methylesterase, CheR-like methyltransferase and BaeS-like histidine kinase; CheR-BaeS |
|  |  |  |  | JN757_14020 |  |
|  |  |  |  | JN757_21030 |  |
|  |  |  |  | JN757_14035 | Sensory box histidine kinase/response regulator |
|  |  |  |  | JN757_14040 |  |
|  |  |  |  | JN757_14015 | Two-component system sensor histidine kinase/response hybrid |
|  |  |  |  | JN757_14030 |  |
|  |  |  |  | JN757_08710 | Two-component system, chemotaxis family, response regulator; CheY |
|  |  |  |  | JN757_10240 |  |
|  |  |  |  | JN757_06330 | Chemotaxis protein methyltransferase; (EC:2.1.1.80); CheR |
|  |  |  |  | JN757_14020 |  |
|  |  |  |  | JN757_21030 | Purine-binding chemotaxis protein; CheW |
|  |  |  |  | JN757_06335 |  |
|  |  |  |  | JN757_26345 |  |
|  |  |  |  | JN757_02800 | Methyl-accepting chemotaxis protein; MCPs |
|  |  |  |  | JN757_02895 |  |
|  |  |  |  | JN757_03235 |  |
|  |  |  |  | JN757_03665 |  |
|  |  |  |  | JN757_04130 |  |
|  |  |  |  | JN757_04150 |  |
|  |  |  |  | JN757_04350 |  |
|  |  |  |  | JN757_04640 |  |
|  |  |  |  | JN757_04985 |  |
|  |  |  |  | JN757_06320 |  |
|  |  |  |  | JN757_09030 |  |
|  |  |  |  | JN757_11185 |  |
|  |  |  |  | JN757_13785 |  |
|  |  |  |  | JN757_14640 |  |
|  |  |  |  | JN757_14785 |  |
|  |  |  |  | JN757_14865 |  |
|  |  |  |  | JN757_14925 |  |
|  |  |  |  | JN757_14925 |  |
|  |  |  |  | JN757_15900 |  |
|  |  |  |  | JN757_16100 |  |
|  |  |  |  | JN757_17340 |  |
|  |  |  |  | JN757_17600 |  |
|  |  |  |  | JN757_18810 |  |
|  |  |  |  | JN757_18820 |  |
|  |  |  |  | JN757_19945 |  |
|  |  |  |  | JN757_20735 |  |
|  |  |  |  | JN757_21390 |  |
|  |  |  |  | JN757_21500 |  |
|  |  |  |  | JN757_21675 |  |
|  |  |  |  | JN757_21925 |  |
|  |  |  |  | JN757_23370 |  |
|  |  |  |  | JN757_23590 |  |
|  |  |  |  | JN757_23640 |  |
|  |  |  |  | JN757_23990 |  |
|  |  |  |  | JN757_26355 |  |
|  |  |  |  | JN757_27335 |  |
|  | **Motility and biofilm formation** | **Twitching motility/ adherent proteins** | **Type IV pili biosynthesis** | JN757_23925 | Type IV pilus assembly protein; PilA |
|  |  |  |  | JN757_23930 | Type IV pilus assembly protein; PilB |
|  |  |  |  | JN757_26940 |  |
|  |  |  |  | JN757_23935 | type IV pilus assembly protein; PilC |
|  |  |  |  | JN757_24070 | type IV pilus assembly protein; PilE |
|  |  |  |  | JN757_22795 | type IV pilus assembly protein; PilF |
|  |  |  |  | JN757_26370 | twitching motility two-component system response regulator; PilG |
|  |  |  |  | JN757_26365 | twitching motility two-component system response regulator; PilH |
|  |  |  |  | JN757_26360 | twitching motility protein CheW type; PilI |
|  |  |  |  | JN757_26355 | twitching motility protein; PilJ |
|  |  |  |  | JN757_14520 | type IV pilus assembly protein; PilL |
|  |  |  |  | JN757_03030 | type IV pilus assembly protein; PilM |
|  |  |  |  | JN757_03030 | type IV pilus assembly protein; PilN |
|  |  |  |  | JN757_03040 | type IV pilus assembly protein; PilO |
|  |  |  |  | JN757_03045 | type IV pilus assembly protein; PilP |
|  |  |  |  | JN757_03050 | type IV pilus assembly protein; PilQ |
|  |  |  |  | JN757_24045 | two-component system, NtrC family, response regulator; PilR |
|  |  |  |  | JN757_24040 | Two-component sensor; PilS |
|  |  |  |  | JN757_13505 | twitching motility protein; PilT |
|  |  |  |  | JN757_26425 |  |
|  |  |  |  | JN757_24075 | type IV pilus assembly protein; PilV |
|  |  |  |  | JN757_24070 | type IV pilus assembly protein; PilW |
|  |  |  |  | JN757_24065 | type IV pilus assembly protein; PilX |
|  |  |  |  | JN757_24060 | type IV pilus assembly protein; PilY1 |
|  |  |  |  | JN757_20500 | type IV pilus biogenesis protein; PilZ |
|  | **Biofilm formation** | **Fimbrial adherent proteins** | **Tig adherence (Tad) pilus assembly proteins** | JN757_04315 | Flp pilus assembly protein; CpaA |
|  |  |  |  | JN757_04310 | Flp pilus assembly protein; CpaB |
|  |  |  |  | JN757_04305 | Flp pilus assembly protein; CpaC |
|  |  |  |  | JN757_04300 | Flp pilus assembly protein; CpaE |
|  |  |  |  | JN757_04295 | Flp pilus assembly protein; CpaF |
|  |  |  |  | JN757_04285 | tight adherence protein C; TadC |
|  |  |  |  | JN757_04290 | tight adherence protein D; TadD |
|  |  |  |  | JN757_04260 | tight adherence protein G; TadG |
|  |  |  | **Adherence (Tad) pilus structural proteins** | JN757_24080 | Type IV fimbrial biogenesis protein; FimU |
|  |  |  |  | JN757_24085 | Type IV fimbrial biogenesis protein; FimT |
|  |  |  |  | JN757_08240 | Type IV fimbrial biogenesis protein; FimV |
|  |  | **EPS synthesis** | **PGA synthesis** | JN757_01905 | biofilm PGA synthesis lipoprotein; PgaA [EC:3.-.-.-] |
|  |  |  |  | JN757_01910 | biofilm PGA synthesis protein; PgaB |
|  |  |  |  | JN757_01915 | biofilm PGA synthesis N-glycosyltransferase; PgaC [EC:2.4.-.-] |
|  |  |  |  | JN757_01920 | biofilm PGA synthesis protein; PgaD |
|  |  |  | **Alginate biosynthesis** | JN757_01355 | Alginate biosynthesis two-component system response regulator; AlgB |
|  |  |  |  | JN757_01360 | Alginate biosynthesis two-component system sensor histidine kinase; KinB |
|  |  |  |  | JN757_05775 | Alginate O-acetyltransferase, periplasmic; AlgF |
|  |  |  |  | JN757_05780 | Alginate O-acetyltransferase, inner membrane; AlgJ |
|  |  |  |  | JN757_05790 | Alginate lyase (EC 4.2.2.3); AlgL |
|  |  |  |  | JN757_05795 | Alginate O-acetyltransferase, periplasmic; AlgX |
|  |  |  |  | JN757_05800 | Poly (beta-D-mannuronate) C5 epimerase (EC 5.1.3.-); AlgG |
|  |  |  |  | JN757_05805 | Alginate export system Algk/AlgE, outer membrane porin; AlgE |
|  |  |  |  | JN757_05810 | Alginate export system AlgK/AlgE, periplasmic component; AlgK |
|  |  |  |  | JN757_05815 | Alginate polymerisation protein, membrane fusion protein; Alg44 |
|  |  |  |  | JN757_05820 | Alginate polymerase/glycosyltransferase; Alg8 |
|  |  |  |  | JN757_05825 | GDP-mannose 6-dehydrogenase (EC 1.1.1.132); AlgD |
|  |  |  |  | JN757_05935 | Transcriptional regulatory protein (positive transcriptional regulator of AlgD); AlgP |
|  |  |  |  | JN757_27180 | Alginate regulatory protein, positive transcriptional regulator of AlgD; AlgP |
|  |  |  |  | JN757_27190 | Alginate regulatory protein, positive transcriptional regulator of AlgD; AlgQ |
|  |  |  |  | JN757_27220 | Alginate biosynthesis two-component system response regulator; AlgR |
|  |  |  |  | JN757_21140 | Alginate biosynthesis transcriptional activator |

| **PLANT GROWTH PROMOTION** | **Phosphorous and potassium solubilisation** | **Organic phosphate dephosphorylation enzymes** | **Organic compound dephosphorylation enzymes** | JN757_24790 | Inorganic pyrophosphatase (EC 3.6.1.1) |
| --- | --- | --- | --- | --- | --- |
|  |  |  |  | JN757_25785 | Putative phosphatase (BLAST: PhoX family phosphatase); alkaline phosphatase; PhoX |
|  |  |  |  | JN757_05015 | Phosphodiesterase/alkaline phosphatase D; PhoD |
|  |  |  |  | JN757_27100 | Exopolyphosphatase (EC 3.6.1.11) |
|  |  |  |  | JN757_27105 | Polyphosphate kinase (EC 2.7.4.1); ppk |
|  |  |  |  | JN757_24165 | C-P lyase (alkylphosphonate utilization protein); PhnA |
|  |  |  |  | JN757_16265 | C-P lyase protein; PhnB |
|  |  |  | **Phosphorous solubilization regulatory components** | JN757_00485 | Phosphate regulon transcriptional regulatory protein (SphR); PhoB |
|  |  |  |  | JN757_00490 | Phosphate regulon sensor protein (SphS) (EC 2.7.13.3); PhoR |
|  |  |  |  | JN757_00495 | Phosphate regulon metal ion transporter containing CBS domains |
|  |  |  |  | JN757_00505 | Phosphate regulon transcriptional regulatory protein (SphR); PhoB |
|  |  |  |  | JN757_00515 | Phosphate transport system regulatory protein; PhoU |
|  |  |  | **Phosphorous compounds transporters** | JN757_00515 | Phosphate ABC transporter, ATP-binding protein (TC 3.A.1.7.1); PstB |
|  |  |  |  | JN757_00520 | Phosphate ABC transporter, permease protein (TC 3.A.1.7.1); PstA |
|  |  |  |  | JN757_00525 | Phosphate ABC transporter, permease protein (TC 3.A.1.7.1); PstC |
|  |  |  |  | JN757_00530 | Phosphate ABC transporter, substrate-binding protein (TC 3.A.1.7.1); PstS |
|  |  | **Inorganic phosphorous solubilisation** | **Organic acid synthesis enzymes and co-factors** | JN757_25805 | Malate synthase G (EC 2.3.3.9) |
|  |  |  |  | JN757_01540 | Gluconate 2-dehydrogenase (EC 1.1.99.3), membrane-bound, cytochrome c |
|  |  |  |  | JN757_01550 | Gluconate 2-dehydrogenase (EC 1.1.99.3), membrane-bound, flavoprotein |
|  |  |  |  | JN757_04490 | Malonate-semialdehyde dehydrogenase [inositol] (EC 1.2.1.18) |
|  |  |  |  | JN757_13795 | 2-ketogluconate 6-phosphate reductase (EC 1.1.1.43) |
|  |  |  |  | JN757_13800 | 2-ketogluconate transporter |
|  |  |  |  | JN757_13805 | 2-ketogluconate kinase (EC 2.7.1.13) |
|  |  |  |  | JN757_20890 | D-lactate dehydrogenase (EC 1.1.1.28) |
|  |  |  |  | JN757_08925 | Citrate synthase (si) (EC 2.3.3.1); GltA |
|  |  |  |  | JN757_22645 | Glucose dehydrogenase, PQQ-dependent (EC 1.1.5.2). |
|  |  |  |  | JN757_25675 | Coenzyme PQQ synthesis protein F; PqqF |
|  |  |  |  | JN757_25680 | Coenzyme PQQ synthesis protein B; PqqB |
|  |  |  |  | JN757_25685 | Coenzyme PQQ synthesis protein A; PqqA |
|  |  |  |  | JN757_25690 | Pyrroloquinoline-quinone synthase (EC 1.3.3.11); PqqC |
|  |  |  |  | JN757_25695 | Coenzyme PQQ synthesis protein D; PqqD |
|  |  |  |  | JN757_25700 | Coenzyme PQQ synthesis protein E; PqqE |
|  |  |  |  | JN757_25745 | Coenzyme PQQ synthesis protein E; PqqE |
|  |  |  |  | JN757_25690 | Pyrroloquinoline quinone (Coenzyme PQQ) biosynthesis protein C; PqqC |
|  | **Iron uptake** | **Siderophore synthesis** | **Pyoverdine biosynthesis and transport** | JN757_19545 | Siderophore synthetase small component, acetyltransferase; PvdY/PvdZ |
|  |  |  |  | JN757_19540 | Sigma factor; PvdS |
|  |  |  |  | JN757_19535 | NRPS; Pioverdine chromophore precursor synthethase; PvdA/PvdL |
|  |  |  |  | JN757_19490 | DaT/ L-2,4-diaminobutyrate:2-oxoglutarate aminotransferase; PvdH |
|  |  |  |  | JN757_19485 | MbtH-like NRPS chaperone |
|  |  |  |  | JN757_19470 | ABC transporter in pyoverdin gene cluster, ATP-binding component |
|  |  |  |  | JN757_19475 | ABC transporter in pyoverdin gene cluster, permease component |
|  |  |  |  | JN757_19480 | ABC transporter in pyoverdin gene cluster, periplasmic component |
|  |  |  |  | JN757_19465 | Cation ABC transporter, periplasmic cation-binding protein |
|  |  |  |  | JN757_19460 | FIG049111: Hypothetical protein in pyoverdin gene cluster |
|  |  |  |  | JN757_19455 | FIG137877: Hypothetical protein in pyoverdin gene cluster |
|  |  |  |  | JN757_19450 | FIG139991: Putative thiamine pyrophosphate-requiring enzyme |
|  |  |  |  | JN757_19445 | FIG137594: Putative iron-regulated membrane protein |
|  |  |  |  | JN757_10150 | Putative sterol carrier protein |
|  |  |  |  | JN757_10155 | Thioesterase involved in non-ribosomal peptide biosynthesis |
|  |  |  |  | JN757_10160 | Non-ribosomal peptide synthetase modules; PvdI |
|  |  |  |  | JN757_10165 | Non-ribosomal peptide synthetase modules; PvdJ |
|  |  |  |  | JN757_10170 | Esterase/lipase |
|  |  |  |  | JN757_10175 | TonB-dependent siderophore receptors |
|  |  |  |  | JN757_10180 | Pyoverdine ABC export system, fused ATPase and permease components; PvdE |
|  |  |  |  | JN757_10185 | Pyoverdine responsive serine/threonine kinase; PvdO |
|  |  |  |  | JN757_10190 | Aminotransferase; PvdN |
|  |  |  |  | JN757_10195 | Dipeptidase; PvdM |
|  |  |  |  | JN757_10200 | PvdP |
|  |  |  |  | JN757_10205 | RND efflux transporter; Efflux transport system, outer membrane factor (OMF) lipoprotein |
|  |  |  |  | JN757_10210 | ABC efflux transporter; Macrolide export ATP-binding/permease protein; MacB |
|  |  |  |  | JN757_10215 | RND efflux transporter; Macrolide-specific efflux protein; MacA |
|  |  |  |  | JN757_10220 | Sigma-70 factor; FpvI |
|  |  |  |  | JN757_10225 | L-ornithine 5-monooxygenase: PvdA |
|  |  |  |  | JN757_16075 | Acylase; PvdQ |
|  |  |  |  | JN757_16070 | Transmembrane sensor; repressor of PvdS (negative regulator of pyoverdine synthesis); FpvR |
|  | **Plant hormone synthesis** | **IAA (auxin) synthesis** | **Tryptophan synthesis** | JN757_05135 | Tryptophan synthase beta chain like (EC 4.2.1.20); TrpB |
|  |  |  |  | JN757_10415 | Phosphoribosylanthranilate isomerase (EC 5.3.1.24); TrpC |
|  |  |  |  | JN757_01635 | Tryptophan synthase beta chain (EC 4.2.1.20); TrpB |
|  |  |  |  | JN757_01640 | Tryptophan synthase alpha chain (EC 4.2.1.20); TrpA |
|  |  |  |  | JN757_25465 | Indole-3-glycerol phosphate synthase (EC 4.1.1.48); TrpC |
|  |  |  |  | JN757_25470 | Anthranilate phosphoribosyltransferase (EC 2.4.2.18); TrpD |
|  |  |  |  | JN757_25470 | Anthranilate synthase, amidotransferase component (EC 4.1.3.27) Amidotransferase component (EC 2.6.1.85); TrpE |
|  |  |  | **IAA synthesis** | JN757_22450 | Tryptophan 2-monooxygenase (EC 1.13.12.3); IaaM |
|  |  |  |  | JN757_22455 | Indoleacetamide hydrolase (EC 3.5.1.-); IaaH |
|  | **Plant osmotic stress alleviation** | **Spermidine biosynthesis** | | JN757_04195 | Biosynthetic arginine decarboxylase (EC 4.1.1.19); SpeA​ |
|  |  |  |  | JN757_02465 | Agmatine deiminase (EC 3.5.3.12); AguA​ |
|  |  |  |  | JN757_02460 | N-carbamoylputrescine amidase (EC 3.5.1.53); AguB​ |
|  |  |  |  | JN757_05050 | Pyridoxal 5-phosphate (PLP)-dependent ornithine decarboxylase (EC 4.1.1.17); Odc​ |
|  |  |  |  | JN757_05055 | Osmoprotectant ABC transporter, ATP-binding protein; OsmV​ |
|  |  |  |  | JN757_05060 | Osmoprotectant ABC transporter, permease protein; OsmW​ |
|  |  |  |  | JN757_05065 | Osmoprotectant ABC transporter, substrate-binding protein; OsmX​ |
|  |  |  |  | JN757_05070 | Osmoprotectant ABC transporter, permease protein; OsmY​ |
|  |  |  |  | JN757_25445 | S-adenosylmethionine decarboxylase proenzyme (EC 4.1.1.50), prokaryotic class 1A; SpeD |
|  |  |  |  | JN757_09560 | Spermidine synthase (EC 2.5.1.16); SpeE​ |
|  |  |  |  | JN757_16320 | Spermidine synthase long (EC 2.5.1.16); SpeE​ |
|  |  |  |  | JN757_12010 | Polyamine ABC transporter, ATP-binding protein​ |
|  |  |  |  | JN757_12015 | Polyamine ABC transporter, permease protein​ |
|  |  |  |  | JN757_12020 | Polyamine ABC transporter, permease protein​ |
|  |  |  |  | JN757_12025 | Spermidine/putrescine-binding periplasmic protein; PotD​ |
|  |  |  |  | JN757_25505 | Spermidine/putrescine import ABC transporter permease protein (TC 3.A.1.11.1)​; PotC |
|  |  |  |  | JN757_25510 | Spermidine/putrescine import ABC transporter permease protein (TC 3.A.1.11.1)​; PotB |
|  |  |  |  | JN757_25515 | Spermidine/putrescine import ABC transporter substrate-binding protein (TC 3.A.1.11.1)​; PotD |
|  |  |  |  | JN757_25520 | Spermidine/putrescine import ABC transporter ATP-binding protein (TC 3.A.1.11.1)​; PotA |
|  |  |  |  | JN757_25525 | DNA-binding response regulator, LuxR family |
|  |  |  |  | JN757_26830 | Putrescine transport system permease protein (TC 3.A.1.11.2); PotI |
|  |  |  |  | JN757_26825 | Putrescine transport system permease protein (TC 3.A.1.11.2); PotH |
|  |  |  |  | JN757_26835 | Putrescine transport ATP-binding protein (TC 3.A.1.11.2); PotG |
|  |  |  |  | JN757_26840 | Putrescine ABC transporter putrescine-binding protein (TC 3.A.1.11.2); PotF |
|  |  |  |  | JN757_26845 | Putrescine ABC transporter putrescine-binding protein (TC 3.A.1.11.2); PotF |
|  |  |  |  | JN757_10490 | Gamma-glutamyl-putrescine oxidase (EC1.4.3.-)​ |
|  |  |  |  | JN757_00025 | Putrescine utilization regulator​ |
|  |  | **Trehalose synthesis** | | JN757_12795 | Trehalose synthase (EC 5.4.99.16); TreS |
|  |  |  |  | JN757_12800 | Maltodextrin glucosidase (EC 3.2.1.20); MalZ |
|  |  |  |  | JN757_16130 | Malto-oligosyltrehalose trehalohydrolase (EC 3.2.1.141); TreZ |
|  |  |  |  | JN757_16135 | 4-alpha-glucanotransferase (amylomaltase) (EC 2.4.1.25), MalQ |
|  |  |  |  | JN757_16140 | Malto-oligosyltrehalose synthase (EC 5.4.99.15); TreY |
|  |  |  |  | JN757_16190 | 1,4-alpha-glucan (glycogen) branching enzyme, GH-13-type (EC 2.4.1.18); GlgB |
|  |  |  |  | JN757_16195 | Trehalose synthase (EC 5.4.99.16); TreS |
|  |  |  |  | JN757_16200 | Alpha-amylase (EC 3.2.1.1), alpha-1,4-glucan-maltose-1-phosphate maltosyltransferase |
|  |  |  |  | JN757_22700 | Trehalose-6-phosphate hydrolase (EC 3.2.1.93); TreC |
|  |  |  |  | JN757_22705 | PTS system, trehalose-specific IIB/C component (EC 2.7.1.201); TreP |
|  |  |  |  | JN757_22710 | Trehalose operon transcriptional repressor; TreR |
|  | **Biocontrol molecules** | **HCN biosynthesis** | | JN757_17720 | Hydrogen cyanide synthase, Opine oxidase subunit B; HcnC |
|  |  |  |  | JN757_17715 | Hydrogen cyanide synthase, Opine oxidase subunit A; HcnB |
|  |  |  |  | JN757_17710 | Hydrogen cyanide synthase, Opine oxidase subunit C; HcnA |
|  |  | **Pyocin domain containing proteins** | | JN757_06735 | pyocin R2_PP, holin (Homolog to *P. fluorescens* Pf0-1; PP_01352) |
|  |  |  |  | JN757_06905 | pyocin R2_PP, lytic enzyme (glycoside hydrolase family 19 protein) |
|  |  |  |  | JN757_12885 | hypothetical protein (S-type pyocin domain-containing protein) |
|  |  |  |  |  | Uropathogenic specific protein (S-type pyocin domain-containing protein) |
|  |  |  |  | JN757_05570 | S-type pyocin domain-containing protein |
|  |  |  |  | JN757_07230 | S-type pyocin domain-containing protein |
|  |  | **Phenazyne synthesis** | | JN757_22390 | Phenazine biosynthesis protein PhzF like |
|  |  | **Insecticidal proteins** | | JN757_02170 | Putative insecticidal toxin complex |
|  |  |  |  | JN757_05765 | Putative insecticidal toxin complex |
|  |  |  |  | JN757_05970 | Putative insecticidal toxin complex |
|  |  |  |  | JN757_18105 | Putative toxin subunit |
|  |  |  |  | JN757_18110 | putative insecticidal toxin |
|  |  |  |  | JN757_18115 | Putative insecticidal toxin complex |
|  |  |  |  | JN757_22050 | Putative toxin subunit |
|  |  |  |  | JN757_22055 | putative insecticidal toxin |
|  | **Plant defence priming molecules** | **Lokisin biosynthesis AntiSMASH predicted genes** | | JN757_17055 | Alkene reductase, NADH:flavin oxidoreductases |
|  |  |  |  | JN757_17060 | Cryptochrome/photolyase family protein |
|  |  |  |  | JN757_17065 | PAS domain-containing protein, Sensory box histidine kinase/response regulator |
|  |  |  |  | JN757_17070 | Sensor domain-containing diguanylate cyclase, Two-component transcriptional response regulator, LuxR family |
|  |  |  |  | JN757_17075 | AraC family transcriptional regulator |
|  |  |  |  | JN757_17080 | MFS transporter |
|  |  |  |  | JN757_17085 | Hydrolase |
|  |  |  |  | JN757_17090 | Polysaccharide lyase family 7 protein |
|  |  |  |  | JN757_17095 | General stress protein, alginate lyase |
|  |  |  |  | JN757_17100 | Hypothetical protein |
|  |  |  |  | JN757_17105 | ATP-dependent Clp protease proteolytic subunit |
|  |  |  |  | JN757_17110 | SCO family protein, Cytochrome oxidase biogenesis protein Sco1/SenC/PrrC |
|  |  |  |  | JN757_17115 | Copper metallochaperone PCu(A)C |
|  |  |  |  | JN757_17120 | Hypothetical protein |
|  |  |  |  | JN757_17125 | Fic family protein, PA1366 type |
|  |  |  |  | JN757_17130 | Helix-turn-helix transcriptional regulator |
|  |  |  |  | JN757_17135 | Family efflux pump subunit; MacB |
|  |  |  |  | JN757_17140 | Macrolide transporter subunit; MacA |
|  |  |  |  | JN757_17145 | Amino acid adenylation domain-containing protein |
|  |  |  |  | JN757_17150 | Amino acid adenylation domain-containing protein |
|  |  |  |  | JN757_17155 | Amino acid adenylation domain-containing protein |
|  |  |  |  | JN757_17160 | Helix-turn-helix transcriptional regulator |
|  |  |  |  | JN757_17165 | Efflux transporter outer membrane subunit |
|  |  |  |  | JN757_17170 | ABC transporter substrate-binding protein; TauA |
|  |  |  |  | JN757_17175 | LTA synthase family protein |
|  |  |  |  | JN757_17180 | Catalase/peroxidase HPI (EC 1.11.1.21); KatG |
|  |  |  |  | JN757_17185 | Type I methionyl aminopeptidase (EC 3.4.11.18) |
|  |  |  |  | JN757_17190 | ParD-like family protein |
|  |  |  |  | JN757_17195 | ABC transporter substrate-binding protein |
|  |  |  |  | JN757_17200 | Iron ABC transporter permease |
|  |  |  |  | JN757_17205 | ABC transporter ATP-binding protein |
|  |  |  |  | JN757_17210 | Pyridoxal-phosphate dependent enzyme |
|  |  |  |  | JN757_17215 | Cystathionine gamma-synthase |
|  |  |  |  | JN757_17220 | Response regulator |
|  |  |  |  | JN757_17225 | Response regulator transcription factor |
|  |  |  |  | JN757_17230 | Fe^2+^ Zn^2+^ uptake regulation protein |
| **SURVIVAL IN THE RHIZOSPHERE** | **Metabolic versatility** | **Substrate utilisation** | **Xylulose utilisation** | JN757_12815 | D-xylose ABC transporter, permease protein; XylH |
|  |  |  |  | JN757_12820 | D-xylose ABC transporter, ATP-binding protein; XylG |
|  |  |  |  | JN757_12825 | D-xylose ABC transporter, substrate-binding protein; XylF |
|  |  |  |  | JN757_12820 | Xylose isomerase (EC 5.3.1.5); XylA |
|  |  |  |  | JN757_12835 | Xylose activator (AraC family), XylR |
|  |  |  |  | JN757_13960 | Xylulose kinase (EC 2.7.1.17) |
|  |  |  |  | JN757_15580 | Xylose isomerase-like TIM-barrel protein KPN_00539 |
|  |  |  | **N-acetylglucosamine utilisation** | JN757_06050 | PTS system, N-acetylglucosamine-specific IIC component / PTS system, N-acetylglucosamine-specific IIB component (EC 2.7.1.193) |
|  |  |  |  | JN757_06055 | PTS system, N-acetylglucosamine-specific IIA component (EC 2.7.1.193);PtsP |
|  |  |  |  | JN757_06060 | Glucosamine-6-phosphate deaminase [isomerizing], alternative (EC 3.5.99.6) |
|  |  |  |  | JN757_06065 | N-acetylglucosamine-6-phosphate deacetylase (EC 3.5.1.25) |
|  |  |  |  | JN757_06070 | Predicted transcriptional regulator of N-Acetylglucosamine utilization, GntR family |
|  |  | **Storage molecules** | **Polyhydroxyalkanoate biosynthesis** | JN757_02950 | Polyhydroxyalkanoate granule-associated protein; PhaI |
|  |  |  |  | JN757_02955 | Polyhydroxyalkanoate granule-associated protein; PhaF |
|  |  |  |  | JN757_02960 | Transcriptional regulator (Ter-like); PhaD |
|  |  |  |  | JN757_02965 | Polyhydroxyalkanoic acid synthase; PhaC |
|  |  |  |  | JN757_02970 | Poly(3-hydroxyalkanoate) depolymerase; PhaZ |
|  |  |  |  | JN757_02975 | Polyhydroxyalkanoic acid synthase; PhaC |
|  |  |  | **Cyanophycin biosynthesis** | JN757_20270 | Cyanophycin synthase (EC 6.3.2.29) (EC 6.3.2.30); CphA |
|  |  |  |  | JN757_20275 | Cyanophycinase 2 (EC 3.4.15.6); CphB |
|  |  |  |  | JN757_20280 | Asparagine synthetase [glutamine-hydrolyzing] (EC 6.3.5.4) |
|  | **Abiotic stress tolerance** | **Ionic stress alleviation** | **Potassium uptake** | JN757_00725 | Putative Glutathione-regulated potassium-efflux system protein; KefB |
|  |  |  |  | JN757_01185 | Trk potassium uptake system protein; TrkA |
|  |  |  |  | JN757_07000 | Kup system potassium uptake protein, Kup |
|  |  |  |  | JN757_08290 | Trk potassium uptake system protein; TrkH |
|  |  |  |  | JN757_14145 |  |
|  |  |  |  | JN757_09340 | Potassium efflux system KefA protein / Small-conductance mechanosensitive channel; KefA |
|  |  |  |  | JN757_16475 |  |
|  |  |  |  | JN757_16815 |  |
|  |  |  |  | JN757_25025 |  |
|  |  |  |  | JN757_19975 | DNA-binding response regulator; KdpE |
|  |  |  |  | JN757_19980 | Osmosensitive K+ channel histidine kinase; KdpD |
|  |  |  |  | JN757_19985 | Potassium-transporting ATPase C chain (EC 3.6.3.12) (TC 3.A.3.7.1); KdpC |
|  |  |  |  | JN757_19990 | Potassium-transporting ATPase B ch; ain (EC 3.6.3.12) (TC 3.A.3.7.1); KdpB |
|  |  |  |  | JN757_19995 | Potassium-transporting ATPase A chain (EC 3.6.3.12) (TC 3.A.3.7.1); KdpA |
|  |  | **Compatible solute accumulation** | **Glycine betaine metabolism** | JN757_25945 | Glycine betaine demethylase subunit B; GbcB |
|  |  |  |  | JN757_25950 | Glycine betaine demethylase subunit A; GbcA |
|  |  |  |  | JN757_25965 | Dimethylglycine demethylase subunit B; DgcB |
|  |  |  |  | JN757_25970 | Dimethylglycine demethylase subunit A; DgcA |
|  |  |  | **Choline/betaine metabolism** | JN757_26005 | Choline binding ABC transport system substrate-binding protein; ChoX |
|  |  |  |  | JN757_01245 | Choline binding ABC transport system permease protein; ChoW |
|  |  |  |  | JN757_26050 |  |
|  |  |  |  | JN757_26055 | Choline binding ABC transport system ATP-binding protein; ChoV |
|  |  |  |  | JN757_02235 | High-affinity choline uptake protein; BetT |
|  |  |  |  | JN757_26060 |  |
|  |  |  |  | JN757_26065 | Transcriptional regulator (TetR family); BetI |
|  |  |  |  | JN757_26070 | Betaine aldehyde dehydrogenase (EC 1.2.1.8); BetB |
|  |  |  |  | JN757_26075 | Choline dehydrogenase (EC 1.1.99.1); BetA |
|  |  |  |  | JN757_14150 | Secondary glycine betaine transporter; BetU |
|  |  |  |  | JN757_08910 | Betaine ABC transporter, substrate-binding protein; BetX |
|  |  |  |  | JN757_00545 | L-Proline/Glycine betaine transporter; ProP |
|  |  |  |  | JN757_14095 |  |
|  |  |  |  | JN757_23645 |  |
|  |  |  |  | JN757_10130 | Glucoamylase (EC 3.2.1.3) |
|  |  |  | **Trehalose** | JN757_12795 | Trehalose synthase (EC 5.4.99.16); TreS |
|  |  |  |  | JN757_12800 | Maltodextrin glucosidase (EC 3.2.1.20); MalZ |
|  |  |  |  | JN757_16130 | Malto-oligosyltrehalose trehalohydrolase (EC 3.2.1.141); TreZ |
|  |  |  |  | JN757_16135 | 4-alpha-glucanotransferase (amylomaltase) (EC 2.4.1.25) |
|  |  |  |  | JN757_16140 | Malto-oligosyltrehalose synthase (EC 5.4.99.15); TreY |
|  |  |  |  | JN757_16190 | 1,4-alpha-glucan (glycogen) branching enzyme, GH-13-type (EC 2.4.1.18) |
|  |  |  |  | JN757_16195 | Trehalose synthase (EC 5.4.99.16); TreS |
|  |  |  |  | JN757_16200 | Alpha-amylase (EC 3.2.1.1) |
|  |  |  |  | JN757_22700 | Trehalose-6-phosphate hydrolase (EC 3.2.1.93); TreC |
|  |  |  |  | JN757_22705 | PTS system, trehalose-specific IIB component (EC 2.7.1.201) / PTS system, trehalose-specific IIC component; TreP |
|  |  |  |  | JN757_22710 | Trehalose operon transcriptional repressor; TreR |
|  |  | **Cold coping mechanism** | **Cold shock proteins** | JN757_06480 | Cold shock protein of CSP family; CspC |
|  |  |  |  | JN757_07075 | Cold shock protein of CSP family; CspA |
|  |  |  |  | JN757_10590 | Cold shock protein of CSP family; CspG |
|  |  |  |  | JN757_17980 | Cold shock protein of CSP family; CspD |
|  |  |  |  | JN757_21745 | Cold shock protein of CSP family; CspA |
|  |  |  |  | JN757_21850 | hypothetical domain / Cold shock protein of CSP family; CspA |
|  | **Heavy metal resistance** | **Chromate resistance** | | JN757_11745 | Chromate transport protein; ChrA |
|  |  |  |  | JN757_11965 | Chromate reductase (EC 1.6.5.2); ChrR |
|  |  | **Copper resistance** | | JN757_16105 | CopG |
|  |  |  |  | JN757_18960 | CopD |
|  |  |  |  | JN757_18955 | CopC |
|  |  |  |  | JN757_18950 | CopB |
|  |  |  |  | JN757_18945 | Multicopper oxidase, CopA family copper-resistance protein; CopA |
|  |  |  |  | JN757_19125 | Copper tolerance protein, cupredoxin |
|  |  |  |  | JN757_19130 | Copper-sensing two-component system response regulator; CusR |
|  |  |  |  | JN757_19135 | Copper sensory histidine kinase; CusS |
|  |  |  |  | JN757_04015 | Multicopper oxidases |
|  |  |  |  | JN757_22680 |  |
|  |  |  |  | JN757_04365 | Heavy metal resistance transcriptional regulator HmrR; CueR |
|  |  |  |  | JN757_04370 | Lead, cadmium, zinc and mercury transporting ATPase (EC 3.6.3.3) (EC 3.6.3.5); Copper-translocating P-type ATPase (EC 3.6.3.4); CopA |
|  |  |  |  | JN757_04380 | Copper (I) chaperone, CopZ |
|  |  |  |  | JN757_23110 | DNA-binding heavy metal response regulator |
|  |  |  |  | JN757_04020 | Copper metallochaperone PCu(A)C, inserts Cu(I) into cytochrome oxidase subunit II |
|  |  |  |  | JN757_17115 |  |
|  |  |  |  | JN757_01500 | Cytochrome oxidase biogenesis protein Cox11-CtaG, copper delivery to Cox1 |
|  |  |  |  | JN757_10020 | Type cbb3 cytochrome oxidase biogenesis protein CcoI; Copper-translocating P-type ATPase (EC 3.6.3.4) |
|  |  |  |  | JN757_10005 | Heavy-metal-associated domain (N-terminus) and membrane-bounded cytochrome biogenesis cycZ-like domain, possible membrane copper tolerance protein |
|  |  |  |  | JN757_08810 | Cytochrome c heme lyase subunit; CcmH |
|  |  |  |  | JN757_08800 | Cytochrome c heme lyase subunit; CcmF |
|  |  |  |  | JN757_04385 | Multidrug resistance transporter, Bcr/CflA family |
|  |  |  |  | JN757_16695 |  |
|  |  |  |  | JN757_26610 |  |
|  |  |  |  | JN757_26675 | Lead, cadmium, zinc and mercury transporting ATPase (EC 3.6.3.3) (EC 3.6.3.5); Copper-translocating P-type ATPase (EC 3.6.3.4) |
|  |  |  |  | JN757_24565 | Apolipoprotein N-acyltransferase / Copper homeostasis protein; CutE |
|  |  |  |  | JN757_24560 | Magnesium and cobalt efflux protein CorC (copper homeostasis) |
|  |  | **Cobalt/zinc/cadmium resistance** | | JN757_02020 | Two-component transcriptional response regulator (OmpR family) |
|  |  |  |  | JN757_02025 | Heavy metal sensor histidine kinase |
|  |  |  |  | JN757_04365 | Heavy metal resistance transcriptional regulator; HmrR |
|  |  |  |  | JN757_04370 | Lead, cadmium, zinc and mercury transporting ATPase (EC 3.6.3.3) (EC 3.6.3.5); Copper-translocating P-type ATPase (EC 3.6.3.4) |
|  |  |  |  | JN757_05530 | Transcriptional regulator (MerR family) |
|  |  |  |  | JN757_11765 | Heavy metal sensor histidine kinase |
|  |  |  |  | JN757_11770 | DNA-binding heavy metal response regulator |
|  |  |  |  | JN757_19130 | Copper-sensing two-component system response regulator; CusR |
|  |  |  |  | JN757_19135 | Copper sensory histidine kinase; CusS |
|  |  |  |  | JN757_23100 | RND efflux system, membrane fusion protein |
|  |  |  |  | JN757_23105 | Multidrug efflux system MdtABC-TolC, inner-membrane proton/drug antiporter MdtB-like |
|  |  |  |  | JN757_23110 | DNA-binding heavy metal response regulator |
|  |  |  |  | JN757_23115 | Heavy metal sensor histidine kinase |
|  |  |  |  | JN757_26675 | Lead, cadmium, zinc and mercury transporting ATPase (EC 3.6.3.3) (EC 3.6.3.5); Copper-translocating P-type ATPase (EC 3.6.3.4) |
|  |  |  |  | JN757_26680 | Cd(II)/Pb(II)-responsive transcriptional regulator |
|  |  | **Arsenic resistance** | | JN757_11970 | Arsenite/antimonite:H+ antiporter; ArsB |
|  |  |  |  | JN757_11370 | Arsenical resistance operon repressor; ArsR |
|  |  |  |  | JN757_11375 | Arsenate reductase (EC 1.20.4.4) thioredoxin-coupled, LMWP family; ArsC-2 |
|  |  |  |  | JN757_11380 | Arsenic resistance protein; ArsH |
|  |  |  |  | JN757_20915 | Arsenate reductase (glutarredoxin); ArsC-1 |
|  | **Toxic compound resistance** | **Tabtoxin resistance** | | JN757_03960 | Tabtoxin resistance protein; acetyltransferase |
|  |  |  |  | JN757_22945 | bacteriocin/colicin immunity protein |
|  |  |  |  | JN757_24135 | Conserved uncharacterized protein; CreA |
|  |  |  |  | JN757_25855 | Inner membrane protein; CreD |
|  |  |  |  | JN757_25860 | Sensory histidine kinase CreC of two-component signal transduction system; CreBC |
|  |  |  |  | JN757_25865 | Response regulator CreB of two-component signal transduction system; CreBC |
|  |  | **Macrolide antibiotic resistance** | | JN757_10210 | Macrolide export ATP-binding/permease protein MacB (ABC efflux transporter) |
|  |  |  |  | JN757_10215 | Macrolide-specific efflux protein MacA (RND efflux transporter) |
|  |  | **Fluoroquinolones resistance** | | JN757_01130 | DNA gyrase subunit B (EC 5.99.1.3), GyrB |
|  |  |  |  | JN757_03480 | DNA topoisomerase IV subunit B (EC 5.99.1.3), parB |
|  |  |  |  | JN757_03495 | DNA topoisomerase IV subunit A (EC 5.99.1.3), parC |
|  |  |  |  | JN757_20165 | DNA gyrase subunit A (EC 5.99.1.3), GyrA |
|  |  | **Multidrug efflux system** | | JN757_16240 | Outer membrane factor (OMF) lipoprotein associated wth MdtABC efflux system |
|  |  |  |  | JN757_16245 | Multidrug efflux system MdtABC-TolC, inner-membrane proton/drug antiporter MdtC (RND type); MdtC |
|  |  |  |  | JN757_16250 | Multidrug efflux system MdtABC-TolC, inner-membrane proton/drug antiporter MdtB (RND type); MdtB |
|  |  |  |  | JN757_16255 | Multidrug efflux system MdtABC-TolC, membrane fusion component; MdtA |
|  |  |  |  | JN757_23100 | RND efflux system, membrane fusion protein |
|  |  |  |  | JN757_23105 | Multidrug efflux system MdtABC-TolC, inner-membrane proton/drug antiporter MdtB-like |
|  |  |  |  | JN757_11520 | Inner-membrane proton/drug antiporter (MSF type) of tripartite multidrug efflux system |
|  |  |  |  | JN757_11525 | Membrane fusion component of MSF-type tripartite multidrug efflux system |
|  |  |  |  | JN757_15615 | Multidrug efflux system, membrane fusion component, MexE of MexEF-OprN system; MexE |
|  |  |  |  | JN757_15620 | Multidrug efflux system, inner membrane proton/drug antiporter (RND type), MexF of MexEF-OprN system; MexF |
|  |  |  |  | JN757_15635 | Multidrug efflux system, outer membrane factor lipoprotein, OprN of MexEF-OprN system; OprN |
|  | **Plant defence evasion** | **Plant defence compound detoxification** | **Nitrile detoxification and nitrogen acquisition** | JN757_16035 | Plant-induced nitrilase (EC 3.5.5.1), hydrolyses beta-cyano-L-alanine |
|  |  |  |  | JN757_16040 | Transcriptional regulator in cluster with plant-induced nitrilase |
|  |  |  | **Cyanate detoxification** | JN757_11330 | Cyanate hydratase (EC 4.2.1.104), cyanase, CysS |
|  |  |  |  | JN757_11335 | Cyn operon transcriptional activator, CynR |
|  |  |  | **Nitric oxide free radical detoxification** | JN757_23065 | Flavohemoglobin nitric oxide dioxygenase, induced by Nitric Oxide |
|  |  |  |  | JN757_23070 | Anaerobic nitric oxide reductase transcription regulator; NorR |
|  |  | **Oxidative stress protection** | **ROS detoxification** | JN757_01445 | Catalase KatE-intracellular protease (EC 1.11.1.6); KatE |
|  |  |  |  | JN757_02005 | Transcriptional regulator, Crp/Fnr family |
|  |  |  |  | JN757_05295 | Superoxide dismutase [Mn] (EC 1.15.1.1) |
|  |  |  |  | JN757_09240 | Catalase-like heme-binding protein |
|  |  |  |  | JN757_11180 | Superoxide dismutase [Cu-Zn] precursor (EC 1.15.1.1) |
|  |  |  |  | JN757_11820 | Phytochrome, two-component sensor histidine kinase (EC 2.7.3.-) |
|  |  |  |  | JN757_11825 | Catalase-peroxidase KatG (EC 1.11.1.21); KatG |
|  |  |  |  | JN757_17180 | Catalase-like heme-binding protein |
|  |  |  |  | JN757_18285 | NTP pyrophosphohydrolases including oxidative damage repair enzymes |
|  |  |  |  | JN757_19415 | Redox-sensitive transcriptional activator; SoxR |
|  |  |  |  | JN757_19415 | Organic hydroperoxide resistance transcriptional regulator; SoxR |
|  |  |  |  | JN757_19735 | Organic hydroperoxide resistance transcriptional regulator |
|  |  |  |  | JN757_19820 | Organic hydroperoxide resistance protein |
|  |  |  |  | JN757_19820 | Superoxide dismutase [Fe] (EC 1.15.1.1) |
|  |  |  |  | JN757_22180 | Alkyl hydroperoxide reductase subunit C-like protein |
|  |  |  |  | JN757_22350 | Cytochrome c551 peroxidase (EC 1.11.1.5) |
|  |  |  |  | JN757_22540 | Catalase KatE (EC 1.11.1.6); KatE |
|  |  |  |  | JN757_24290 | Catalase KatB (EC 1.11.1.6); KatB |
|  |  |  |  | JN757_25120 | Alkyl hydroperoxide reductase subunit C-like protein |
|  |  |  |  | JN757_26900 | Alkyl hydroperoxide reductase subunit C-like protein |
|  |  | **Plant immune defence response alleviation** | **GABA catabolism** | JN757_02580 | GABA permease; GabP |
|  |  |  |  | JN757_01945 | Succinate-semialdehyde dehydrogenase [NAD(P)+] (EC 1.2.1.16) |
|  |  |  |  | JN757_01950 | 5-aminovalerate aminotransferase (EC 2.6.1.48) / Gamma-aminobutyrate:alpha-ketoglutarate aminotransferase (EC 2.6.1.19); GabT |
|  |  |  |  | JN757_03805 | gamma-aminobutyric acid ABC transporter, substrate-binding protein |
|  |  |  |  | JN757_03810 | gamma-aminobutyric acid ABC transporter, permease protein 1 |
|  |  |  |  | JN757_03815 | gamma-aminobutyric acid ABC transporter, permease protein 2 |
|  |  |  |  | JN757_03820 | gamma-aminobutyric acid ABC transporter, ATP-binding protein 1 |
|  |  |  |  | JN757_03825 | gamma-aminobutyric acid ABC transporter, ATP-binding protein 2 |
| **VIRULENCE TRAITS** | **Secretion systems** | **Type VI Secretion System (T6SS)** | **T6SS structural genes** | JN757_00320 | VgrG protein; VgrG |
|  |  |  |  | JN757_00325 | T6SS Serine/threonine protein kinase (EC 2.7.11.1); PpkA |
|  |  |  |  | JN757_00330 | T6SS protein serine/threonine phosphatase; PppA |
|  |  |  |  | JN757_00335 | T6SS component tssM (IcmF/VasK); TssM |
|  |  |  |  | JN757_00340 | T6SS outer membrane component TssL (ImpK/VasF); TssL |
|  |  |  |  | JN757_00345 | T6SS component TssK (ImpJ/VasE); TssK |
|  |  |  |  | JN757_00350 | T6SS secretion lipoprotein TssJ (VasD); TssJ |
|  |  |  |  | JN757_00355 | T6SS forkhead associated domain protein ImpI/VasC, tagH (type VI secretion system-associated FHA domain protein); TagH |
|  |  |  |  | JN757_00360 | FIG00959590: hypothetical protein (Type VI secretion protein) |
|  |  |  |  | JN757_00365 | T6SS sigma-54-dependent regulator; VasH |
|  |  |  |  | JN757_00370 | T6SS AAA+ chaperone ClpV; TssH |
|  |  |  |  | JN757_00375 | T6SS component (ImpH/VasB); TssG |
|  |  |  |  | JN757_00380 | T6SS component (ImpG/VasA); TssF |
|  |  |  |  | JN757_00385 | hypothetical protein |
|  |  |  |  | JN757_00390 | hypothetical protein |
|  |  |  |  | JN757_00395 | hypothetical protein |
|  |  |  |  | JN757_00400 | T6SS PAAR-repeat protein |
|  |  |  |  | JN757_00405 | T6SS lysozyme-like component; TssE |
|  |  |  |  | JN757_00410 | T6SS component (ImpC/VipB); TssC |
|  |  |  |  | JN757_00415 | T6SS component (ImpB/VipA); TssB |
|  |  |  |  | JN757_00420 | T6SS component (ImpA); TssA |
|  |  |  |  | JN757_00425 | Hypothetical protein |
|  |  |  |  | JN757_00430 | VgrG protein; VgrG |
|  |  |  |  | JN757_00435 | Hypothetical protein |
|  |  |  |  | JN757_00440 | Lipoprotein |
|  |  |  |  | JN757_00445 | Lipase; TseL |
|  |  |  | **T6SS effector genes** | JN757_01280 | T6SS PAAR-repeat protein |
|  |  |  |  | JN757_03270 | VgrG protein; VgrG |
|  |  |  |  | JN757_04170 | T6SS component; Hcp |
|  |  |  |  | JN757_04200 | VgrG protein; VgrG |
|  |  |  |  | JN757_06275 | T6SS PAAR-repeat protein; RhaS protein |
|  |  |  |  | JN757_06280 | T6SS PAAR-repeat protein; RhaS protein |
|  |  |  |  | JN757_06285 | hypothetical protein |
|  |  |  |  | JN757_06290 | T6SS PAAR-repeat protein; RhaS protein |
|  |  |  |  | JN757_06295 | Hypothetical protein |
|  |  |  |  | JN757_06300 | T6SS PAAR-repeat protein; RhaS protein |
|  |  |  |  | JN757_06305 | FIG00963848: hypothetical protein |
|  |  |  |  | JN757_06310 | VgrG protein; VgrG |
|  |  |  |  | JN757_06315 | T6SS component; Hcp |
|  |  |  |  | JN757_09275 | T6SS PAAR-repeat protein; RhaS protein |
|  |  |  |  | JN757_12290 | T6SS PAAR-repeat protein |
|  |  |  |  | JN757_13075 | VgrG protein; VgrG |
|  |  |  |  | JN757_13080 | T6SS component; Hcp |
|  |  |  |  | JN757_13945 | VgrG protein; VgrG |
|  |  |  |  | JN757_14250 | T6SS component; Hcp |
|  |  |  |  | JN757_14255 | VgrG protein; VgrG |
|  |  |  |  | JN757_14260 | Rhs-family protein |
|  |  |  |  | JN757_14275 | T6SS PAAR-repeat protein |
|  |  |  |  | JN757_14280 | Rhs-family protein |
|  |  |  |  | JN757_17635 | T6SS component; Hcp |
|  |  |  |  | JN757_18330 | VgrG protein; VgrG |
|  |  |  |  | JN757_18755 | VgrG protein; VgrG |
|  |  |  |  | JN757_18930 | T6SS PAAR-repeat protein |

**Table S2. Plant specificity and ecological annotation of *P. granadensis* CT364 methyl-accepting protein (MCP) chemoreceptor genes.** The table provides the following information: the MCP accession number from the CT364 genome, the MCP from the Sanchis *et al*., database with the highest similarity, and the similarity between the two; the ‘Degree of Plant Specificity (DPS)’ from the corresponding ligand binding domains (LBD) group; and the ecological information retrieved from the best-hit from the MCP in the Genome of Earth’s Microbiome (GEM) catalogue. Red shading indicates an identity lower than 80%. Blue shading indicates a DPS greater than 50%. Green shading indicates that the MCP is predicted to be present in the ‘Plant’ and ‘Rhizoplane’ ecosystems.

| MCP accession in CT364 genome | MCP best-hit from Sanchis *et al*., database (taxID.biosample.Protein_accesion) | % identity between them | DPS for the LBD | Ecological information retrieved from the best-hit in GEM catalogue |
| --- | --- | --- | --- | --- |
| JN757_RS01755 | 294.SAMN04558174.GCA_001648775_05248 | 98.562 | 36.73 | [‘Built environment’; ‘City’; ‘city subway metal’] |
| JN757_RS02800 | 294.SAMN05860393.GCA_001878715_04826 | 99.206 | 23.32 | [‘Plants’; ‘Rhizoplane’; ‘Arabidopsis rhizosphere’] |
| JN757_RS02895 | 294.SAMN05860393.GCA_001878715_04805 | 98.746 | 64.63 | [‘Plants’; ‘Rhizoplane’; ‘Arabidopsis rhizosphere’] |
| JN757_RS03235 | 294.SAMN05860393.GCA_001878715_04737 | 97.412 | 67.12 | [‘Built environment’; ‘City’; ‘city subway metal’] |
| JN757_RS03665 | 294.SAMN05860393.GCA_001878715_04650 | 99.689 | 43.67 | [‘Built environment’; ‘City’; ‘city subway metal’] |
| JN757_RS04130 | 323656.SAMEA2698475.NGAL_HAMBI1146_46490 | 24.627 | 30.1 | [‘Built environment’; ‘City’; ‘city subway wood’] |
| JN757_RS04150 | 294.SAMN05860393.GCA_001878715_04553 | 97.913 |  | [‘Plants’; ‘Rhizoplane’; ‘Arabidopsis rhizosphere’] |
| JN757_RS04350 | 294.SAMN04558174.GCA_001648775_04377 | 98.148 | 18.03 | [‘Plants’; ‘Rhizoplane’; ‘Arabidopsis rhizosphere’] |
| JN757_RS04640 | 294.SAMN05860393.GCA_001878715_04468 | 98.279 | 53.98 | [‘Built environment’; ‘City’; ‘city subway metal’] |
| JN757_RS04985 | 294.SAMN05860393.GCA_001878715_04413 | 97.041 | 39.02 | [‘Built environment’; ‘City’; ‘city subway metal’] |
| JN757_RS06320 | 294.SAMN05860393.GCA_001878715_04156 | 95.37 | 43.37 | [‘Plants’; ‘Rhizoplane’; ‘Arabidopsis rhizosphere’] |
| JN757_RS09035 | 294.SAMN05860393.GCA_001878715_03589 | 97.782 | 46.67 | [‘Plants’; ‘Rhizoplane’; ‘Arabidopsis rhizosphere’] |
| JN757_RS11195 | 294.SAMN05860393.GCA_001878715_03301 | 93.217 | 41.57 | [‘Built environment’; ‘City’; ‘city subway metal’] |
| JN757_RS13795 | 294.SAMN05860393.GCA_001878715_02911 | 97.043 | 49.09 | [‘Plants’; ‘Rhizoplane’; ‘Arabidopsis rhizosphere’] |
| JN757_RS14650 | 543360.SAMN05216600.SAMN05216600_102220 | 53.429 | 17.85 | [‘Aquatic’; ‘Freshwater’; ‘Freshwater Sediment’] |
| JN757_RS14795 | 1940241.SAMN06275483.BZK31_04075 | 67.837 | 56.25 | [‘Plants’; ‘Rhizosphere’; ‘Root nodules’] |
| JN757_RS14875 | 1674920.SAMN03785348.ACR52_20795 | 67.506 | 38.33 | [‘Built environment’; ‘City’; ‘city subway metal’] |
| JN757_RS14935 | 294.SAMN03398829.VO64_5532 | 59.184 |  | [‘Built environment’; ‘City’; ‘city subway metal’] |
| JN757_RS14935 | 294.SAMN04094107.AO356_21915 | 83.333 | 40 | [‘Plants’; ‘Rhizoplane’; ‘Arabidopsis rhizosphere’] |
| JN757_RS15910 | 294.SAMN05860393.GCA_001878715_02706 | 98.713 | 34.68 | [‘Built environment’; ‘City’; ‘city subway metal’] |
| JN757_RS16110 | 294.SAMN05860393.GCA_001878715_02658 | 99.698 | 37.31 | [‘Plants’; ‘Rhizoplane’; ‘Arabidopsis rhizosphere’] |
| JN757_RS17350 | 294.SAMN05860393.GCA_001878715_03173 | 99.228 | 19.88 | [‘Plants’; ‘Rhizoplane’; ‘Arabidopsis rhizosphere’] |
| JN757_RS17610 | 294.SAMN05860393.GCA_001878715_03228 | 96.629 | 72.73 | [‘Plants’; ‘Rhizoplane’; ‘Arabidopsis rhizosphere’] |
| JN757_RS18820 | 294.SAMN05860393.GCA_001878715_02009 | 99.255 | 46.05 | [‘Plants’; ‘Rhizosphere’; ‘Miscanthus rhizosphere’] |
| JN757_RS18830 | 205922.SAMN02598267.Pfl01_3770 | 95.245 |  | [‘Built environment’; ‘City’; ‘city subway metal’] |
| JN757_RS19955 | 294.SAMN05860393.GCA_001878715_01721 | 95.538 | 61.11 | [‘Plants’; ‘Rhizosphere’; ‘Miscanthus rhizosphere’] |
| JN757_RS20745 | 294.SAMN04558174.GCA_001648775_04718 | 95.115 | 51 | [‘Plants’; ‘Rhizoplane’; ‘Arabidopsis rhizosphere’] |
| JN757_RS21375 | 294.SAMN05860393.GCA_001878715_01441 | 99.317 | 22.52 | [‘Aquatic’; ‘Marine’; ‘Deep oceanic; basalt-hosted subsurface hydrothermal fluid’] |
| JN757_RS21400 | 294.SAMN04558174.GCA_001648775_03412 | 93.704 | 35.8 | [‘Plants’; ‘Rhizoplane’; ‘Arabidopsis rhizosphere’] |
| JN757_RS27570 | 294.SAMN05860393.GCA_001878715_01412 | 99.544 | 38.33 | [‘Built environment’; ‘City’; ‘city subway metal’] |
| JN757_RS21685 | 294.SAMN05860393.GCA_001878715_01376 | 98.736 | 17.85 | [‘Built environment’; ‘City’; ‘city subway metal’] |
| JN757_RS21935 | 294.SAMN05860393.GCA_001878715_01325 | 98.092 | 18.97 | [‘Plants’; ‘Rhizoplane’; ‘Arabidopsis rhizosphere’] |
| JN757_RS23380 | 294.SAMN05860393.GCA_001878715_01018 | 98.457 | 17.85 | [‘Plants’; ‘Rhizoplane’; ‘Arabidopsis rhizosphere’] |
| JN757_RS23600 | 294.SAMN05860393.GCA_001878715_00973 | 97.226 | 62.82 | [‘Built environment’; ‘City’; ‘city subway metal’] |
| JN757_RS27585 | 294.SAMN05860393.GCA_001878715_00966 | 97.945 | 30.1 | [‘Built environment’; ‘City’; ‘city subway metal’] |
| JN757_RS24000 | 294.SAMN05860393.GCA_001878715_00897 | 97.967 | 64.62 | [‘Plants’; ‘Rhizoplane’; ‘Arabidopsis rhizosphere’] |
| JN757_RS26365 | 294.SAMN05860393.GCA_001878715_00419 | 100 | 42.22 | [‘Aquatic’; ‘Marine’; ‘Deep oceanic; basalt-hosted subsurface hydrothermal fluid’] |
| JN757_RS27345 | 294.SAMN05860393.GCA_001878715_00218 | 99.392 | 30.52 | [‘Built environment’; ‘City’; ‘city subway metal’] |

**Table S3. Plant growth promoting and pathogenic pseudomonad species pan-genomic gene family categorisation.** The list includes the number of the species conserved (core), non-essential (accessory), and exclusive (unique) genes.

| **Strain** | **No. of core genes** | **No. of accessory genes** | **No. of unique genes** | **No. of exclusively absent genes** |
| --- | --- | --- | --- | --- |
| *P. aeruginosa* PAO1 | 2301 | 1476 | 1575 | 278 |
| *P. fluorescens* Pf-01 | 2301 | 2794 | 385 | 1 |
| *P. fluorescens* SBW25 | 2301 | 2833 | 624 | 14 |
| *P. koreensis* LMG 21318 | 2301 | 2648 | 316 | 0 |
| *P. koreensis* S150 | 2301 | 2673 | 466 | 18 |
| *P. moraviensis* LMG 24280 | 2301 | 2496 | 296 | 13 |
| *P. protegens* Pf-5 | 2301 | 2730 | 1006 | 18 |
| *P. putida* BIRD-1 | 2301 | 2179 | 466 | 17 |
| *P. putida* KT2440 | 2301 | 2364 | 588 | 6 |
| *P. putida* W619 | 2301 | 2034 | 643 | 23 |
| *P. simiae* WCS417 | 2301 | 2669 | 421 | 4 |
| *P. granadensis* CT364 | 2301 | 2606 | 420 | 5 |
| *P. granadensis* LMG27940 | 2301 | 2548 | 144 | 4 |

**Table S4. Arabidopsis fresh plant weight (mg) following inoculation with *P. granadensis* CT364.** Plants were grown under soil-mimicking conditions for 28 days before measurement was done. Values showed represent mean ± standard deviation.

|  | **Saline** | **Control** |
| --- | --- | --- |
| **CT364** | 416.41 ±120.49 | 605.43 ±170.86 |
| **Mock** | 311.80 ±128.75 | 485.94 ±184.83 |

**Table S5. Antimicrobial activity and mode of action screening for *P. granadensis* strain CT364.**

| **Strain** | ***B. subtills* zone of inhibition**  **(mm)** | **LacZ activity^a^** | | | | |
| --- | --- | --- | --- | --- | --- | --- |
|  |  | *dinB* | *yjax* | *ypuA* | *yvgS* | *yvgI* |
| CT364 | 1 | - | + | - | - | - |

*a* Presence of a blue ring around the zone of inhibition + = present with strong colour, - = no blue ring present.
